# Hemodialysis versus peritoneal dialysis in elderly ESRD patients: a retrospective cohort study on survival and the role of comorbidities

**DOI:** 10.1186/s12882-026-04783-7

**Published:** 2026-01-30

**Authors:** Ruizhi Yu, Yunfei Yan, Junjie Lin, Hucai Li, Youqing Cai, Lixin Wang, Xusheng Liu, Zhiren He

**Affiliations:** 1 The Second Clinical College of Guangzhou, University of Chinese Medicine, Guangzhou, Guangdong 510000 China; 2 Department of Nephrology, The Second Affiliated Hospital of Guangzhou, University of Chinese Medicine, Guangzhou, Guangdong 510000 China

**Keywords:** Hemodialysis, Peritoneal dialysis, Selection of dialysis modality, Cause of death, Prognosis, Elderly

## Abstract

**Objective:**

To compare survival differences between hemodialysis (HD) and peritoneal dialysis (PD) in elderly patients (≥ 60 years old) with end-stage renal disease (ESRD), and to analyze the impact of key comorbidities (heart failure, cerebrovascular disease, diabetes) on prognosis and causes of death, to inform dialysis modality selection.

**Methods:**

This single-center retrospective cohort study enrolled 377 elderly ESRD patients (216 HD, 161 PD) who initiated dialysis between 2012 and 2017, with follow-up until 2024. Survival rates were compared using the Kaplan-Meier method. The Cox proportional hazards model was used to analyze risk factors for all-cause mortality, and the competing risks model was employed to assess risks for specific causes of death. Subgroup analyses were performed for patients with heart failure, cerebrovascular disease, and diabetes.

**Results:**

In the overall population, the HD group had a significantly lower risk of all-cause mortality than the PD group (adjusted HR = 0.599, 95% CI: 0.45–0.79). Subgroup analyses revealed that the survival advantage of HD was particularly pronounced in patients with concomitant heart failure (adjusted HR = 0.422, 95% CI: 0.25–0.70) or diabetes (adjusted HR = 0.687, 95% CI: 0.48–0.98). Notably, HD was identified as an independent risk factor for hemorrhagic death (sHR = 8.773, 95% CI: 1.03–74.42), with the PD group demonstrating a markedly lower cumulative incidence of such events.

**Conclusion:**

For elderly ESRD patients, dialysis modality selection should be individualized based on comorbidities. HD may be associated with a survival benefit and might be prioritized for patients with concomitant heart failure or diabetes. PD may be considered a safer option, particularly for patients with cerebrovascular disease who are at high risk of bleeding.

**Clinical trial number:**

Not applicable.

**Supplementary Information:**

The online version contains supplementary material available at 10.1186/s12882-026-04783-7.

## Introduction

### Research background

Against the backdrop of accelerating global population aging, the incidence of ESRD continues to rise.In China, this trend is particularly pronounced: the proportion of individuals aged ≥ 60 years rose from 7.54% in 1982 to 21.07% in 2023, and those aged ≥ 65 years increased from 4.91% to 15.38% [1]. Epidemiological data show that the number of registered dialysis patients in China reached 282,000 in 2012, with an annual growth rate as high as 20–30% [2]. For elderly patients with ESRD, hemodialysis (HD) and peritoneal dialysis (PD) are the mainstay renal replacement therapies. The selection of the optimal modality in this population is complex, challenged by age-related physiological decline, a high burden of comorbidities, and reduced tolerance to treatment-related stressors [3–5]. Among comorbidities, heart failure (HF), cerebrovascular disease (CVD), and diabetes mellitus (DM) are highly prevalent in elderly ESRD patients and profoundly impact prognosis. Preliminary data suggest significant differences in the distribution and prognostic impact of these conditions between HD and PD patients, highlighting their potential critical role in guiding modality selection. While prior studies have compared HD and PD outcomes [6, 7], they often provide limited generalizable evidence for the elderly. More importantly, there is a notable lack of comprehensive, stratified analyses examining outcome differences across specific high-risk comorbidity subgroups (e.g., HF, CVD, DM) within this aging population. This gap limits the ability to tailor dialysis therapy based on a patient’s specific comorbid profile.

### Research significance

This study seeks to provide an evidence base for individualized dialysis modality selection in elderly ESRD patients. Theoretically, by elucidating outcome heterogeneity across key comorbidity subgroups, it aims to clarify the interactions between dialysis modality and compromised organ systems (e.g., cardiac function, cerebrovascular integrity). Clinically, it endeavors to establish comorbidity-oriented selection criteria and stratified management protocols, offering concrete guidance for decision-making in this vulnerable population and informing future strategies to optimize dialysis therapy.

## Method

We conducted a retrospective cohort study. Elderly patients with ESRD who visited the Department of Nephrology at Guangdong Provincial Hospital of Chinese Medicine between January 1, 2012, and December 31, 2017, were enrolled. All patients had renal failure due to primary renal diseases (such as chronic glomerulonephritis, diabetic nephropathy, hypertensive nephropathy, etc.) and received maintenance PD or HD treatment. The follow-up end date was June 30, 2024. Data were extracted from the electronic medical record system and the dialysis registry database. The study adhered to the following inclusion and exclusion criteria: **Inclusion Criteria**: (1) Diagnosed with ESRD; (2) Undergoing hemodialysis or peritoneal dialysis for ≥ 3 months; (3) Age ≥ 60 years at initiation of renal replacement therapy; **Exclusion Criteria**: (1) Patients with pre-dialysis malignancies; (2) Patients with baseline data missingness: defined as lacking any core variable for this study, including dialysis modality, dialysis initiation date, age, or primary comorbidities (heart failure, cerebrovascular disease, diabetes); (3) Patients receiving combined renal replacement therapy (simultaneous hemodialysis and peritoneal dialysis); (4) Patients who had received other dialysis therapy for > 3 months prior to inclusion. Patients with less than 3 months of dialysis experience will be excluded. The clinical rationale for this criterion is that the initial 3 months following dialysis initiation are typically regarded as a high mortality risk period or adaptation phase. Mortality during this period is often associated with pre-existing end-stage uremic conditions, severe complications, or acute clinical events at the start of dialysis, rather than the long-term effects of the dialysis modality itself. Collected data included general information (sex, age, primary disease type, and comorbidities), laboratory indicators (serum creatinine, blood urea nitrogen, electrolytes, metabolic indicators such as blood glucose and lipids, inflammation and nutritional markers (C-reactive protein, albumin), cardiac function parameters such as left ventricular ejection fraction), and endpoint events (all-cause death). Data were obtained from the electronic medical record system of Guangdong Provincial Hospital of Traditional Chinese Medicine. Laboratory indicators were uniformly tested according to the standards of the hospital’s laboratory department. Endpoint events were confirmed by medical records and death certificates. All variables in the HD group and PD group were collected using the same data source, testing standards, and assessment procedures to ensure consistency in the evaluation methods. Missing data were filled in using a multiple imputation by chained equations algorithm by using R’s MICE package. Regarding the handling of patients who discontinued the initially assigned dialysis modality or were lost to follow-up, the following events were treated as censoring events in the survival analysis: switching to another dialysis modality, receiving a kidney transplant, transferring to another center for continued treatment, or reaching the end of the study follow-up period (June 30, 2024). Data from these patients contributed to the analysis until the date of censoring. In statistical analysis, continuous variables with normal distribution are presented as mean ± standard deviation and compared using the independent samples t-test; those with non-normal distribution are presented as median (interquartile range) and compared using the Mann-Whitney U test. Categorical variables are presented as frequency (percentage) and compared using the Chi-square test. Overall survival was estimated using the Kaplan-Meier method, and differences between groups were assessed with the Log-rank test. A multivariable Cox proportional hazards model was employed to identify independent prognostic factors. Variables for inclusion in the multivariable model followed the principle of clinical priority supplemented by statistical significance: First, regardless of statistical significance, gender, age at dialysis initiation, and key comorbidities (diabetes, coronary heart disease, cerebrovascular disease history) were mandatorily included. Subsequently, laboratory indicators with *p* < 0.1 in univariate analysis were incorporated into the model. Multicollinearity among covariates was assessed using the variance inflation factor (VIF), with values < 5 considered acceptable (Table S3). The same variable selection principle was applied in the Fine-Gray competing risks regression model, which was used to analyze three specific causes of death (hemorrhagic, cerebrovascular, cardiovascular disease death), considering deaths from other causes as competing events. Specifically: (1) for hemorrhagic death, the competing events were non-hemorrhagic deaths; (2) for cerebrovascular death, the competing events were deaths from non-cerebrovascular causes; (3) for cardiovascular death, the competing events were deaths from non-cardiovascular causes. The same competing risk definitions were applied in subgroup analyses. Finally, the survival analyses (Kaplan-Meier and Log-rank), Cox regression analyses (univariate and multivariate), and competing risks analyses were repeated within predefined subgroups (with comorbid heart failure/cerebrovascular disease/diabetes).Statistical analysis was performed using Python (version 3.12.7; packages: lifelines 0.30.0, scipy 1.13.1) and R (version 4.5.0; package: cmprsk 2.2.12) software packages. Results are presented as hazard ratio (HR) or subdistribution hazard ratio (sHR) with 95% confidence intervals (CI) and p-values. A p-value < 0.05 was considered statistically significant. Additionally, to quantify the clinical benefit of HD over PD, we calculated the Absolute Risk Difference (ARD) and Number Needed to Treat (NNT) based on 5-year survival rates. ARD was defined as the difference in survival probability between HD and PD groups (ARD = HD survival rate - PD survival rate). NNT, representing the number of patients needed to treat with HD instead of PD to prevent one additional death over 5 years, was derived as the reciprocal of the absolute value of ARD (NNT = 1 / |ARD|), with ARD expressed as a decimal (e.g., an ARD of 9.21% was converted to 0.0921 for calculation). These measures were computed using Kaplan-Meier survival estimates at 5 years for populations where survival analysis demonstrated a significant advantage for HD over PD (including the overall population and comorbidity subgroups such as heart failure and diabetes). This study was approved by the Ethics Committee of Guangdong Provincial Hospital of Chinese Medicine (Approval Number: ZE2020-054-01). Patient data were de-identified. The study was conducted in accordance with the ethical principles of the Declaration of Helsinki.

## Results analysis

### Baseline characteristic analysis

**Fig. 1:**
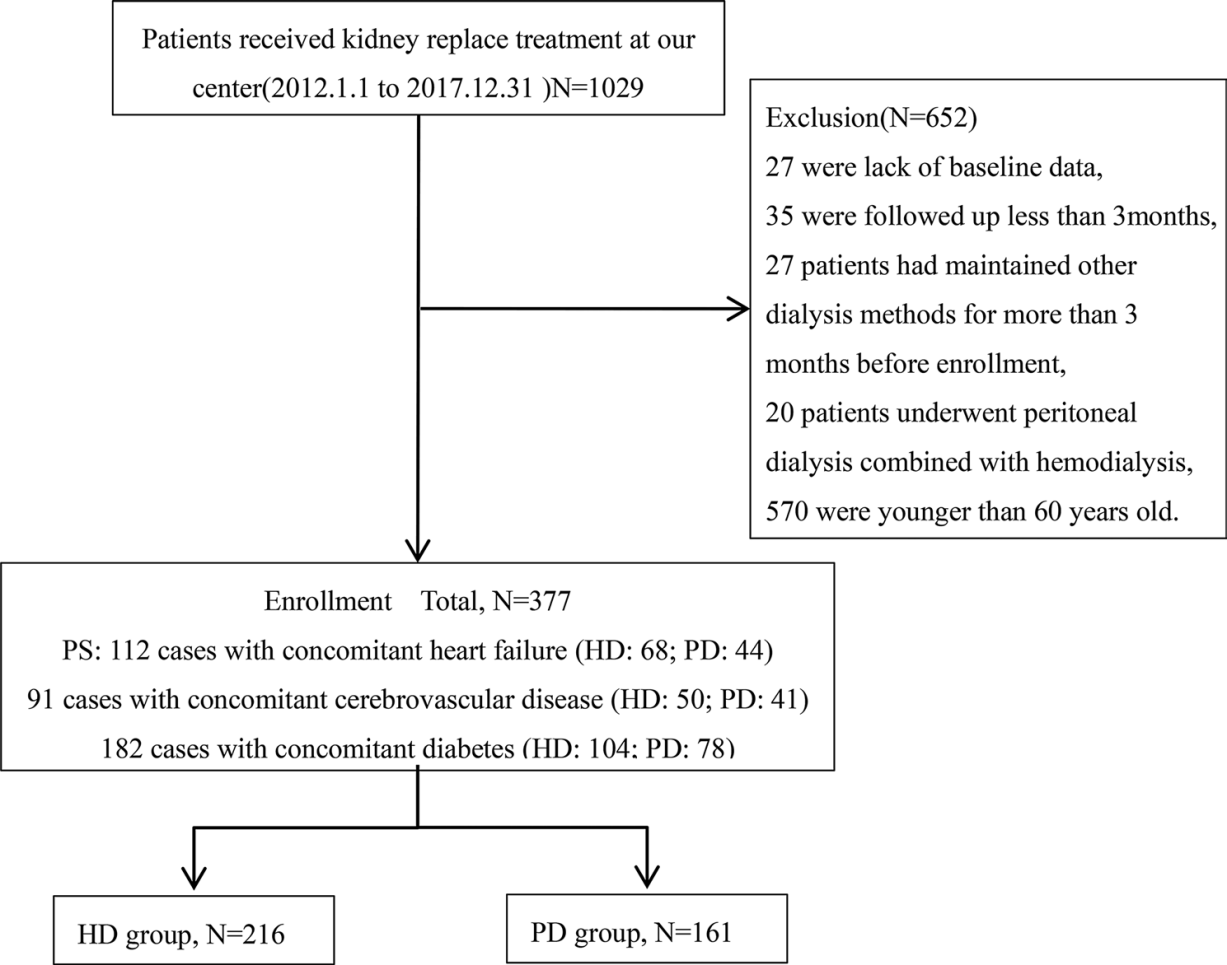
Study flow diagraph

From 1,029 patients receiving kidney replacement therapy (1 January 2012 to 31 December 2017), this study ultimately selected 377 cases ( including 216 in the HD group and 161 in the PD group ) for analysis. Primary exclusion criteria included age non-compliance (*n* = 570), followed by missing baseline data (*n* = 27), dialysis duration < 3 months (*n* = 35), prior dialysis treatment exceeding 3 months before enrollment (*n* = 27), and combined dialysis therapy (*n* = 20) (Fig. 1). Baseline analysis showed (Table 1) that compared with the PD group, patients in the HD group had an older age at dialysis initiation (69 years vs. 67 years, *p* < 0.05) and a longer follow-up duration (1794.23 ± 1054.33 days vs. 1386.74 ± 915.35 days, *p* < 0.05), and a higher proportion of males (53.2% vs. 38.5%, *p* < 0.05). However, no significant differences were observed in the distribution of major comorbidities (e.g., diabetes mellitus, coronary heart disease). Regarding laboratory indicators, the PD group had higher pre-dialysis serum creatinine and plasma prealbumin levels, while the HD group had higher total bilirubin and blood neutrophil counts.

**Table 1:** Comparison of baseline characteristics between the HD group and the PD group

Variables	Overall situation (*n* = 377)	HD group (*n* = 216)	PD group (*n* = 161)	*P*
**Demographic data**				
Male(n)	177(46.9)	115(53.2)	62(38.5)	< 0.05
All-cause death(n)	244(64.7)	143(66.2)	101(62.7)	0.556
follow-up duration(days)	1620.21 ± 1016.32	1794.23 ± 1054.33	1386.74 ± 915.35	< 0.05
Age of dialysis initiation (years)	67.00(64.00–74.00)	69.00(64.00–77.00)	67.00(64.00–72.00)	< 0.05
**Kidney primary disease**				
Glomerulonephritis(n)	101(26.8)	39(18.1)	62(38.5)	< 0.05
Diabetic nephropathy(n)	125(33.2)	64(29.6)	61(37.9)	0.115
Obstructive nephropathy(n)	27(7.2)	13(16.0)	14(8.7)	0.426
Polycystic kidney(n)	11(2.9)	8(3.7)	3(1.9)	0.459
Other(n)	113(30.0)	92(42.6)	21(13.0)	< 0.05
**Comorbidities**				
CCI	7.00(6.00–9.00)	8.00(6.00–9.00)	7.00(6.00–8.00)	0.15
Diabetes (n)	182(48.3)	104(48.1)	78(48.4)	1
Hypertension(n)	359(95.2)	203(94.0)	156(96.9)	0.286
Coronary heart disease(n)	90(23.9)	47(21.8)	43(26.7)	0.321
Cerebrovascular disease(n)	100(26.5)	61(28.2)	39(24.2)	0.45
Chronic pulmonary disease(n)	36(9.5)	20(9.3)	16(9.9)	0.964
Mild liver disease(n)	100(26.5)	57(26.4)	43(26.7)	1
Rheumatism(n)	9(2.4)	3(1.4)	6(3.7)	0.259
Peripheral vascular disease(n)	65(17.2)	35(16.2)	30(18.6)	0.631
Hyperlipidemia(n)	90(23.9)	54(25.0)	36(22.4)	0.636
**Laboratory tests**				
Serum potassium(mmol/L)	4.52 ± 0.81	4.52 ± 0.82	4.51 ± 0.80	0.909
Serum sodium(mmol/L)	140.04 ± 3.88	139.99 ± 3.90	140.09 ± 3.87	0.813
Serum chloride(mmol/L)	101.76 ± 4.99	102.19 ± 4.93	101.19 ± 5.02	0.054
Serum magnesium(mmol/L)	0.89(0.81–0.94)	0.89(0.89–0.94)	0.88(0.74–0.92)	<0.001
Serum calcium(mmol/L)	2.07(1.96–2.17)	2.05(1.96–2.17)	2.08(1.96–2.18)	0.612
Serum phosphorusg(mmol/L)	1.68(1.34–1.97)	1.69(1.31–1.94)	1.64(1.37-2.00)	0.883
PTH(pg/mL)	313.60(167.30-474.90)	359.20(182.47-482.07)	271.60(145.20-470.80)	0.219
TCO_2_(mmol/L)	22.97 ± 4.58	22.68 ± 4.64	23.35 ± 4.49	0.159
Serum glucose(mmol/L)	5.81(4.79–7.52)	6.11(4.93–7.68)	5.47(4.77–7.28)	0.611
Glycosylated hemoglobin(%)	5.85(5.60–6.10)	5.85(5.70–6.03)	5.85(5.40–6.20)	0.599
Serum urea(mmol/L)	22.64 ± 10.69	21.92 ± 10.92	23.62 ± 10.34	0.127
Serum creatinine (µmol/L)	724.43 ± 281.11	694.02 ± 291.76	765.23 ± 261.53	0.015
Serum uric acid(µmol/L)	450.40(361.00-510.00)	455.00(346.75–491.50)	449.90(388.00-519.00)	0.218
Triglyceride(mmol/L)	1.20(0.83–1.59)	1.31(0.89–1.61)	1.08(0.78–1.58)	0.067
Cholesterol(mmol/L)	4.46(3.73–5.02)	4.50(3.81–4.99)	4.32(3.58–5.03)	0.486
LDLC(mmol/L)	2.71(2.10–3.13)	2.75(2.11–3.07)	2.64(2.08–3.27)	0.803
HDLC(mmol/L)	1.06(0.85–1.27)	1.09(0.87–1.19)	1.02(0.84–1.32)	0.769
Plasma albumin (g/L)	34.74 ± 4.94	34.94 ± 5.06	34.47 ± 4.79	0.366
Prealbumin(mg/L)	271.40 ± 80.37	260.18 ± 77.54	286.46 ± 81.87	0.002
Aspartate aminotransferase(U/L)	18.80(13.0-23.40)	19.00(13.00–24.00)	18.50(13.00–23.00)	0.673
Alkaline phosphatase(/L)	72.00(56.00–91.00)	74.00(61.00–91.00)	69.00(54.00–92.00)	0.094
Total bilirubin(µmol/L)	5.40(4.00-7.20)	5.95(4.20–7.72)	5.00(4.00-6.30)	0.004
Blood white blood cell count(×10^9^/L)	6.76(5.16–8.20)	6.89(5.39–8.43)	6.60(4.96–7.88)	0.373
Hemoglobin(g/L)	82.00(71.00–97.00)	82.00(70.00-97.50)	82.00(71.00–94.00)	0.272
C-reactive protein(mg/L)	12.60(3.40–25.40)	14.40(6.00-25.12)	8.80(2.60–25.40)	0.576

HD, hemodialysis; PD, peritoneal dialysis; CCI, Charlson Comorbidities Index; PTH, parathyroid hormone; TCO_2_, total carbon dioxide; LDLC, low-density lipoprotein; HDLC, high-density lipoprotein

### Survival difference analysis

**Fig. 2:**
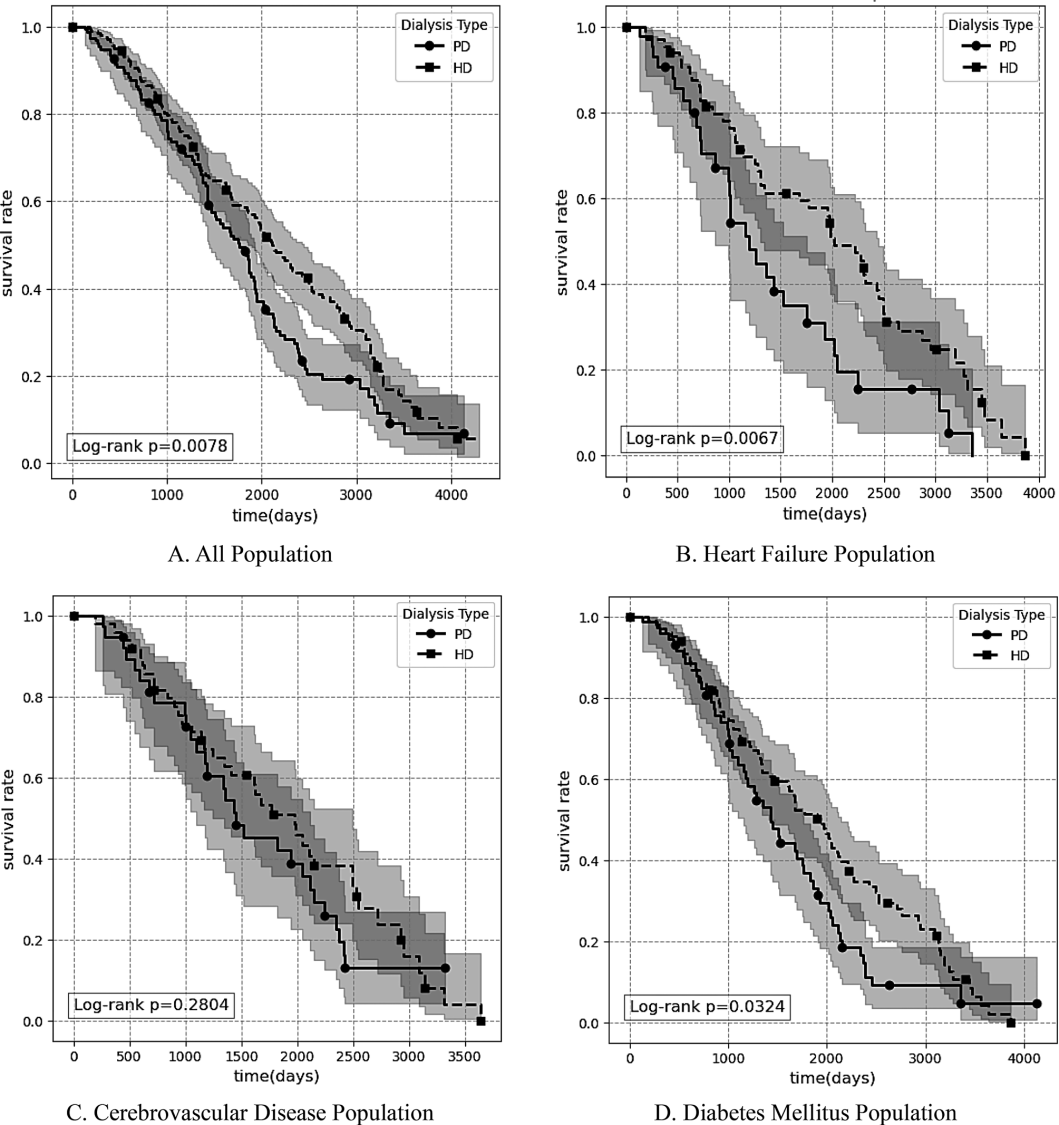
Survival analysis comparison between the HD group and PD group across multiple populations: overall, heart failure, cerebrovascular disease, and diabetes mellitus

This study compared long-term survival outcomes and specific cause-specific mortality risks between HD and PD patients. As shown in Table 2, survival rates at all time points were higher in the HD group than in the PD group across the overall population, with a more pronounced survival advantage at 5 and 10 years (5-year: 58.63% vs. 49.42%; 10-year: 13.02% vs. 6.90%). In the overall population, the survival rate of patients in the HD group was significantly higher than that in the PD group (Log-rank test *p* = 0.0078; Fig. 2A). Based on the 10-year survival rate, the absolute risk difference (ARD) of HD relative to PD was 6.12%, with a number needed to treat (NNT) of 16. This implies that for every 16 elderly ESRD patients who choose HD instead of PD, one death can be prevented within 10 years (Table 3). This survival advantage persisted and was more pronounced in subgroups with concomitant heart failure or diabetes. Among patients with concomitant heart failure, HD therapy demonstrated a significant survival advantage (Log-rank *p* = 0.0067; Fig. 2B). The 10-year survival rate in the HD group was markedly higher than that in the PD group (8.23% vs. 0.00%), with an ARD of 8.23% and an NNT of 12, indicating substantial clinical benefit of HD in this subgroup (Table 3). In the subgroup with comorbid cerebrovascular disease, survival analysis showed (Fig. 2C): there was no statistically significant difference in overall survival between the HD group and the PD group (by the log-rank test, *p* = 0.2804). In the diabetes mellitus subgroup, survival analysis demonstrated higher survival rates in the HD group compared to the PD group (Log-rank *p* = 0.0324; Fig. 2D). The 5-year survival rate in the HD group was 51.26%, superior to the PD group’s 36.81% (Table 2). However, the clinical benefit of HD was not evident in this subgroup when considering 10-year survival rates (ARD 0.31%, NNT 322) (Table 3).This indicates diminishing benefits over the long term. Regarding specific causes of death (Table 4), infectious diseases represented the primary cause of mortality in both groups, with no apparent difference in long-term cumulative incidence between HD and PD (51.13% vs. 45.25%). Nevertheless, a significant intergroup difference emerged in the mortality risk associated with hemorrhagic diseases (including intracerebral hemorrhage and gastrointestinal bleeding). Despite overall low incidence, the cumulative incidence of hemorrhagic mortality was markedly higher in the HD group than in the PD group (15.65% vs. 1.43%), with risk emerging earlier (0.49% at 12 months in the HD group vs. 0% in the PD group at the same time point).

**Table 2:** Comparison of long-term survival between Hemodialysis and peritoneal Dialysis groups in the overall population and key subgroups (heart failure, cerebrovascular disease, and diabetes mellitus)

	Overall situation	HD group	PD group
**All Population**	***n*** = 377	***n*** = 216	***n*** = 161
Median follow-up time(months)	48.03(24.10-81.03)	55.92(30.44–87.65)	44.90(20.50-64.73)
Incidence of death (deaths/1000 person-years)	237.84	223.48	261.65
1-year survival rate(%)	96.41	97.59	94.80
3-year survival rate(%)	76.32	78.23	73.60
5-year survival rate(%)	55.00	58.63	49.42
10-year survival rate(%)	10.68	13.02	6.90
**Heart Failure Population**	***n*** = 112	***n*** = 68	***n*** = 44
Median follow-up time(months)	40.78(20.92–76.84)	57.45(27.34–84.47)	26.48(15.71–48.86)
Incidence of death (deaths/1000 person-years)	267.94	249.79	305.71
1-year survival rate(%)	94.52	97.01	90.58
3-year survival rate(%)	66.45	73.05	54.33
5-year survival rate(%)	48.54	57.73	30.99
10-year survival rate(%)	5.21	8.23	0
**Cerebrovascular Disease Population**	***n*** = 91	***n*** = 50	***n*** = 41
Median follow-up time(months)	47.73(28.05–76.45)	53.58(31.04–83.92)	39.87(22.40-70.57)
Incidence of death (deaths/1000 person-years)	267.27	241.04	313.09
1-year survival rate(%)	96.56	97.96	94.74
3-year survival rate(%)	70.55	71.26	69.44
5-year survival rate(%)	48.74	50.94	45.29
10-year survival rate(%)	5.35	3.97	12.94
**Diabetes Mellitus Population**	***n*** = 182	***n*** = 104	***n*** = 78
Median follow-up time(months)	44.32(23.71–71.50)	52.35(29.47–80.96)	34.27(20.08–60.49)
Incidence of death (deaths/1000 person-years)	279.96	268.21	299.4
1-year survival rate(%)	96.59	97.07	95.94
3-year survival rate(%)	69.65	72.34	65.38
5-year survival rate(%)	45.7	51.26	36.81
10-year survival rate(%)	4.11	4.29	4.6

HD, hemodialysis; PD, peritoneal dialysis

**Table 3:** Comparison of 10-year survival rates, ARD, and NNT between the HD group and the PD group

	HD group 10-year survival rate	PD group 10-year survival rate	ARD	NNT
Overall Population	13.02%	6.9%	6.12%	16
Combined heart failure population	8.23%	0.00%	8.23%	12
Combined cerebrovascular disease population	3.97%	12.94%	8.97%	11
Combined Diabetes Mellitus population	4.29%	4.6%	0.31%	322

ARD, absolute risk difference, ARD = HD group survival rate - PD group survival rate; NNT, the number needed to treat. NNT = 1÷|ARD|

**Table 4:** Comparison of the cumulative incidence of hemorrhagic and infectious disease deaths in the Hemodialysis and peritoneal Dialysis groups

Disease Type and Time Point	Overall situation	HD group	PD group
**Hemorrhagic disease**			
Incident rate	12.61%	15.65%	1.43%
Cumulative 12-month incidence rate	0.28%	0.49%	0.00%
Cumulative 24-month incidence rate	0.88%	1.50%	0.00%
Cumulative 60-month incidence rate	3.36%	4.63%	1.43%
Cumulative 120-month incidence rate	12.61%	15.65%	1.43%
**Infectious disease**			
Incident rate	50.19%	51.13%	45.25%
Cumulative 12-month incidence rate	0.56%	0.00%	1.34%
Cumulative 24-month incidence rate	3.69%	3.14%	4.51%
Cumulative 60-month incidence rate	11.36%	10.59%	12.62%
Cumulative 120-month incidence rate	50.19%	51.13%	45.25%

hemorrhagic disease, inCIuding gastrointestinal hemorrhage and cerebral hemorrhage; HD, hemodialysis; PD, peritoneal dialysis

### Survival analysis and risk factor assessment for mortality in the overall population

To identify factors independently associated with all-cause mortality in the overall population, we performed Cox regression analyses. Multivariate Cox proportional hazards regression analysis, after stepwise adjustment for sex, age at dialysis initiation, comorbidities, nutritional and metabolic indicators, and cardiac function parameters, confirmed HD as an independent protective factor for reducing all-cause mortality risk (Model 5: HR = 0.599, 95% CI: 0.45–0.79). Other independent risk factors included increasing age at dialysis initiation (HR = 1.051 per year increase), concomitant coronary heart disease (HR = 1.673), and higher Charlson Comorbidity Index (HR = 1.097 per point increase). Among metabolic indicators, low albumin (HR = 0.966) and high triglycerides (HR = 1.147) were risk factors, whereas high-density lipoprotein cholesterol (HDL-C) showed a protective effect (HR = 0.640) (Table 5).

Competitive risk modeling for specific causes of death (detailed results in Supplementary Materials Tables S3-S4) revealed risk differences across dialysis modalities. Key results indicate (Table 6) that HD treatment is an independent risk factor for hemorrhagic death. In the multivariable-adjusted model (Model 3), HD treatment was significantly associated with an increased risk of hemorrhagic death compared to PD (HR = 8.773, 95% CI: 1.03–74.42). In contrast, mortality from cardiovascular and cerebrovascular diseases primarily correlates with traditional risk factors such as diabetes, coronary heart disease, dyslipidemia, and cardiac function, showing no significant independent association with dialysis modality itself.

**Table 5:** Univariate to multivariate Cox regression models for the whole population analyzing risk factors for all-cause mortality

	Model1 HR [95%CI]	Model2 HR [95%CI]	Model3 HR [95%CI]	Model4 HR [95%CI]	Model5 HR [95%CI]
Dialysis type(PD vs. HD)	0.706[0.55–0.91]	0.634[0.49–0.83]	0.656[0.50–0.86]	0.612[0.47–0.80]	0.599[0.45–0.79]
Sex(female vs. male)	0.878[0.68–1.13]	0.778[0.6–1.01]	0.744[0.57–0.97]	0.878[0.67–1.15]	0.894[0.68–1.17]
Age at dialysis initiation(per 1 year)	1.047[1.03–1.07]	1.051[1.03–1.07]	1.046[1.03–1.07]	1.051[1.03–1.07]	1.051[1.03–1.07]
BMI(per 1 kg/m2)	0.983[0.95–1.02]	0.985[0.95–1.02]	0.979[0.95–1.01]	0.967[0.93-1.00]	0.971[0.94-1.0]
HBP(per 1mmHg)	0.783[0.42–1.48]	0.717[0.38–1.35]	0.683[0.36–1.29]	0.654[0.34–1.25]	0.72[0.37–1.39]
CAD(Yes vs. No)	1.790[1.36–2.36]	1.673[1.26–2.22]	1.673[1.26–2.22]	1.438[1.07–1.94]	1.315[0.96–1.8]
CCI without age(per 1 point)	1.170[1.09–1.26]	1.151[1.07–1.24]	1.124[1.04–1.22]	1.112[1.03–1.20]	1.098[1.01–1.19]
EF(per 1%)	0.980[0.97–0.99]	0.980[0.97–0.99]	0.985[0.97-1.0]	0.982[0.97–0.99]	0.982[0.97–0.99]
FS(per 1%)	0.969[0.95–0.99]	0.969[0.95–0.99]	0.976[0.96–0.99]	0.973[0.95–0.99]	0.968[0.9–1.04]
Hemoglobin(per 1 g/L)	1.002[1.00-1.01]	0.999[0.99–1.01]	1.001[0.99–1.01]	1.002[1.0-1.01]	1.002[1.0-1.01]
Plasma albumin(per 1 g/L)	0.963[0.94–0.99]	0.958[0.93–0.98]	0.959[0.93–0.98]	0.966[0.94–0.99]	0.966[0.94–0.99]
Total cholesterol(per 1 mmol/L)	0.988[0.88–1.11]	1.048[0.93–1.18]	1.068[0.95–1.2]	1.090[0.97–1.23]	1.101[0.98–1.24]
Triglyceride(per 1 mmol/L)	1.089[0.99–1.20]	1.157[1.05–1.27]	1.147[1.04–1.27]	1.096[0.98–1.22]	1.093[0.98–1.22]
HDLC(per 1 mmol/L)	0.615[0.4–0.95]	0.584[0.38–0.9]	0.650[0.42–1.02]	0.649[0.42–1.01]	0.64[0.41-1.0]
LDLC(per 1 mmol/L)	1.002[0.87–1.16]	1.070[0.93–1.23]	1.093[0.95–1.26]	1.118[0.97–1.29]	1.127[0.98–1.3]
UA(per 1 µmol/L)	0.999[1.0–1.0]	1.000[1.0–1.0]	1.000[1.0–1.0]	1.000[1.0–1.0]	1.000[1.0–1.0]
Phosphorus(per 1 mmol/L)	0.713[0.56–0.91]	0.816[0.64–1.04]	0.879[0.68–1.13]	0.878[0.69–1.12]	0.876[0.69–1.12]
Calcium(per 1 mmol/L)	1.279[0.75–2.17]	1.054[0.6–1.84]	1.028[0.59–1.79]	1.315[0.72–2.39]	1.325[0.74–2.38]

HR, hazard ratio; 95%CI, 95% confidence interval; HD, hemodialysis; PD, peritoneal dialysis; BMI, body mass index; HBP, hypertension; CAD, coronary artery disease; CCI without age, Charlson Comorbidity Index (calculated without age component); EF, left ventricular ejection fraction; FS, left ventricular fractional shortening; HDLC, high-density lipoprotein cholesterol; LDLC, low-density lipoprotein cholesterol; UA, uric acid; Model 1: Univariate analysis; Model 2: Multivariate analysis adjusted for sex and dialysis initiation age; Model 3: Multivariate analysis adjusted for sex, dialysis initiation age and coronary artery disease; Model 4: Multivariate analysis adjusted for sex, dialysis initiation age, CCI without age, serum Plasma albumin, HDLC and serum phosphorus; Model 5: Multivariate analysis adjusted for sex, dialysis initiation age, CCI without age, serum Plasma albumin, HDLC, serum phosphorus, and left ventricular ejection fraction

**Table 6:** Univariate to multivariate competing risk Cox regression models for the overall population analyzing risk factors for hemorrhagic mortality

	Model1 HR [95%CI]	Model2 HR [95%CI]	Model3 HR [95%CI]	Model4 HR [95%CI]
Dialysis type(PD vs. HD)	7.452[0.96–57.56]	9.120[1.1-75.53]	9.076[1.08–76.26]	8.773[1.03–74.42]
Sex(female vs. male)	0.360[0.1–1.31]	0.368[0.1–1.31]	0.375[0.1–1.36]	0.439[0.12–1.58]
Age at dialysis initiation(per 1 year)	0.978[0.9–1.06]	0.986[0.91–1.07]	0.984[0.9–1.07]	0.980[0.9–1.07]
BMI(per 1 kg/m2)	0.956[0.8–1.14]	0.947[0.78–1.16]	0.933[0.74–1.18]	0.939[0.73–1.2]
Diabetes mellitus (Yes vs. No)	1.467[0.47–4.59]	1.414[0.45–4.46]	1.443[0.20–10.2]	1.569[0.21–11.59]
CAD heart(Yes vs. No)	0.971[0.27–3.51]	1.000[0.26–3.78]	0.920[0.25–3.34]	0.856[0.23–3.17]
CCI without age(per 1 point)	1.086[0.82–1.44]	1.077[0.79–1.48]	1.077[0.79–1.48]	1.043[0.76–1.44]
EF(per 1%)	0.992[0.94–1.05]	0.994[0.94–1.05]	0.995[0.94–1.05]	1.000[0.95–1.06]
FS(per 1%)	1.021[0.94–1.10]	1.025[0.95–1.11]	1.027[0.95–1.11]	1.032[0.95–1.12]
Hemoglobin(per 1 g/L)	0.959[0.93–0.99]	0.959[0.93–0.99]	0.960[0.93–0.99]	0.684[0.49–0.96]
Plasma albumin(per 1 g/L)	0.979[0.91–1.05]	0.994[0.92–1.07]	0.997[0.93–1.07]	1.027[0.94–1.12]
Total cholesterol(per 1 mmol/L)	0.993[0.80–1.24]	1.046[0.82–1.33]	1.042[0.81–1.34]	1.178[0.86–1.61]
Triglyceride(per 1 mmol/L)	0.940[0.79–1.11]	1.026[0.84–1.25]	1.005[0.79–1.28]	0.860[0.59–1.26]
HDLC(per 1 mmol/L)	0.083[0.02–0.42]	0.104[0.02–0.50]	0.105[0.02–0.46]	0.806[0.68–0.95]
LDLC(per 1 mmol/L)	1.098[0.88–1.38]	1.102[0.87–1.40]	1.101[0.86–1.41]	1.203[0.86–1.69]
UA(per 1 µmol/L)	1.006[1.00-1.01]	1.005[1.0-1.010]	1.005[1.00-1.01]	1.040[0.99–1.09]
Phosphorus(per 1 mmol/L)	0.971[0.27–3.44]	1.029[0.28–3.77]	1.060[0.28–4.01]	1.071[0.33–3.51]
Calcium(per 1 mmol/L)	0.167[0.02–1.32]	0.152[0.02–1.51]	0.144[0.01–1.51]	0.270[0.03–2.47]

HR, hazard ratio; 95% CI, 95% confidence interval; HD, hemodialysis; PD, peritoneal dialysis; BMI, body mass index; CAD, coronary artery disease; CCI, Charlson Comorbidity Index (calculated without age component); EF, left ventricular ejection fraction; FS, left ventricular fractional shortening; HDLC, high-density lipoprotein cholesterol; LDLC, low-density lipoprotein cholesterol; UA, uric acid; Model 1: Univariate analysis; Model 2: Multivariate analysis adjusted for sex and dialysis initiation age; Model 3: Multivariate analysis adjusted for sex, dialysis initiation age and CCI without age. Model 4: Multivariate analysis adjusted for dialysis initiation age, CCI without age, hemoglobin and HDLD

### Subgroup analysis

#### Heart failure comorbidity subgroup

We further evaluated risk factors for mortality within the heart failure subgroup using Cox and competing risk models. Multivariate Cox regression analysis confirmed HD as an independent protective factor for survival in this subgroup (adjusted HR = 0.422, 95% CI: 0.25–0.70) (Table S6). Competitive risk modeling (Table S7-S9) suggested that hyperuricemia was a risk factor for hemorrhagic and cerebrovascular disease mortality, while elevated serum phosphorus levels may be a protective factor for cerebrovascular disease mortality. Cardiovascular disease mortality risk was primarily associated with higher body mass index (BMI), concomitant coronary heart disease, and elevated low-density lipoprotein cholesterol (LDL-C) levels.

#### Cerebrovascular disease comorbidity subgroup

In the subgroup with comorbid cerebrovascular disease, Multivariate Cox regression analysis adjusted for key clinical indicators suggested that hemodialysis might be a protective factor in this subgroup (Model 5: HR = 0.527, 95% CI: 0.31–0.91), while advanced age was a clear risk factor (Table S10). Competitive risk modeling (Supplementary Materials Tables S10-S12) confirmed that elevated serum uric acid was a consistent risk factor for hemorrhagic and cerebrovascular disease mortality. Furthermore, improved left ventricular function emerged as a key protective factor against cardiovascular disease mortality.

#### Diabetes mellitus comorbidity subgroup

In the diabetes mellitus subgroup, Multivariate Cox regression analysis indicated that HD (HR = 0.687, 95% CI: 0.48–0.98) and higher BMI were protective factors, while concomitant coronary heart disease was an independent risk factor (Table S14). Competitive risk modeling (Tables S14-S16) revealed that elevated HDL-C and triglyceride levels were significant protective factors against hemorrhagic mortality. For cerebrovascular disease mortality, high serum calcium and male sex showed protective effects, though these findings warrant cautious interpretation. Cardiovascular disease mortality risk remained consistently associated with high BMI, history of coronary heart disease, and elevated hemoglobin levels.

## Discussion

This retrospective cohort study systematically compare the long-term survival differences between HD and PD in elderly patients with ESRD, with a focused analysis on the prognostic characteristics of three key comorbidity subgroups: heart failure, cerebrovascular disease, and diabetes mellitus. Our core findings demonstrate a significant survival advantage for HD in the overall population (adjusted HR = 0.599), but this advantage and its underlying drivers exhibited marked heterogeneity across different comorbidity subgroups [8–11]. Dialysis modality selection should therefore be individualized based on comorbidity profiles and risk stratification.

### Mechanisms underlying survival differences by dialysis modality

Our study found that HD was associated with a significant survival advantage even after multivariate adjustment, aligning with some prior reports [12–16], but contrasting with others [17, 18]. This discrepancy may stem from the advanced age and specific comorbidity burden of our cohort. We hypothesize that the survival benefit of HD may be mediated through several potential mechanisms: (1) HD’s more efficient toxin clearance helps improve uremic cardiomyopathy and metabolic acidosis [19, 20]; (2) In-center treatment facilitating closer monitoring and timely management of acute issues such as volume overload and infections [16, 21]; (3) Possibly better control of certain metabolic parameters.Notably, PD was associated with a significantly lower risk of hemorrhagic death than HD (sHR = 8.773). However, it should be emphasized that hemorrhagic death events were very few, leading to an imprecise risk estimate with a wide confidence interval (95% CI: 1.03–74.42). Thus, this result should be interpreted as hypothesis-generating, indicating that PD may reduce bleeding risk potentially by avoiding systemic anticoagulation, yet it does not provide a definitive conclusion. It offers a cautious consideration for dialysis modality selection in patients at high bleeding risk, which warrants confirmation in future large-scale studies.

### Comorbidity-specific effects: HD advantage in heart failure and diabetes subgroups

#### Heart failure subgroup

The survival advantage of HD was most pronounced in patients with heart failure (adjusted HR = 0.422). We postulate that this may primarily relate to superior hemodynamic stability offered by HD through precise, controlled ultrafiltration, reducing cardiac preload and volume stress [6, 16]. In contrast, the sustained intra-abdominal pressure and glucose load from PD may pose additional burdens on compromised cardiac function [22]. This hypothesis suggests that for elderly ESRD patients with significant heart failure, HD should be actively considered, with careful attention to optimizing ultrafiltration protocols.

#### Cerebrovascular disease subgroup

In patients with cerebrovascular disease, we found no statistically significant difference in overall survival between HD and PD. This suggests that neither modality holds an absolute survival advantage for this population, and the choice must be individualized. Importantly, cerebrovascular disease often impairs the motor and cognitive skills required for safe PD [23, 24], leading many such patients in practice to be “non-randomly” selected for HD. This real-world selection bias must be considered when interpreting comparative outcomes. Furthermore, the “non-random” selection of PD patients with better self-care ability (a potential “center” or cultural practice) could bias results against PD if those patients are healthier, making the lack of a PD survival advantage in the CVD subgroup even more notable. A notable secondary finding was the lower early cumulative incidence of hemorrhagic events (0% at 12–24 months) in the PD group versus HD. This may stem from PD avoiding routine systemic anticoagulation and offering more stable hemodynamics, potentially reducing bleeding risk in vulnerable vasculature [25–29]. However, this did not translate into an overall survival benefit, and the small number of specific events( Only 5 deaths from cerebral infarction) limits definitive conclusions. Additionally, the higher baseline neutrophil count in the HD group (4.87 vs. 4.13, *P* = 0.007) suggests a more pronounced chronic inflammatory state, which may accelerate cerebral vascular endothelial damage [30–32].Therefore, in clinical practice, for elderly ESRD patients with cerebrovascular disease who are at very high risk of bleeding (e.g., prior hemorrhagic stroke, requiring long-term anticoagulation) and retain adequate self-care ability, PD may be a safer option.

#### Diabetes subgroup

In the diabetic population, this study found that the overall survival rate of the PD group was significantly lower than that of the HD group, consistent with previous research conclusions [10]. For example, in the multivariate adjusted model, an implausibly high point estimate for cardiovascular death was observed in the PD group (Model 5: HR = 16.701), which, while not statistically reliable due to the wide confidence interval and small number of events, directionally aligns with the overall trend of poorer outcomes for PD in this diabetic subgroup. However, studies from the same center have yielded different conclusions; previous research [33] indicated no significant difference in survival between the two groups in the diabetic population. A key difference is that this study enrolled elderly ESRD patients ≥ 60 years old with a longer follow-up period, which may more readily expose the long-term risk differences of PD in elderly DM patients. The underlying mechanisms for this observed difference may be related to metabolic challenges inherent to PD. It can be hypothesized that long-term exposure to glucose-based dialysate could contribute to metabolic dysregulation, peritoneal membrane alteration, and possibly accelerated vascular injury [34, 35]. Additionally, HD may offer advantages in small-molecule clearance and phosphorus control [35, 36]. Therefore, for elderly ESRD patients with diabetes, proactive management strategies are recommended: mitigating metabolic risk if PD is used, and optimizing lipid profiles along with volume control if HD is chosen.

### Dynamic association between metabolic disorders and prognosis

This study observed distinct metabolic profiles between the two dialysis modalities, which may be associated with prognostic differences. The PD group had a higher baseline prealbumin level, suggesting better initial nutritional status, but this did not translate into a long-term survival advantage. We speculate that this phenomenon may partly stem from adverse metabolic consequences induced by long-term exposure to glucose-containing peritoneal dialysate in PD patients, such as aggravated insulin resistance and dyslipidemia [37]. Multivariate analysis indicated that a higher HDL-C level showed a trend toward being associated with reduced mortality risk, although this association was of borderline statistical significance. Meanwhile, serum triglyceride levels were not independently associated with mortality after full adjustment.In summary, the interpretation of these metabolic indicators requires caution, especially for associations that are weak or based on limited events. Their clinical implications are more hypothesis-generating. Based on this, we suggest that it may be reasonable to adopt differentiated metabolic management strategies tailored to the dialysis modality: HD patients should focus on comprehensive cardiovascular risk management centered on lipid optimization; PD patients need to pay closer attention to metabolic risks related to the glucose load from peritoneal dialysate and strengthen the monitoring and control of blood glucose, lipids, and phosphorus [35, 36].

In addition to survival outcomes, the choice of dialysis modality in elderly patients should also account for patient-centered outcomes such as quality of life (QOL), treatment adherence, and daily functioning. These factors are particularly relevant given the reduced resilience to treatment stressors in this age group. Therefore, a comprehensive assessment integrating both survival benefits and QOL preferences is essential for individualized dialysis planning.

### Strengths and limitations of this study

This study has significant strengths in the research field of dialysis modality selection for elderly ESRD patients. Firstly, the research is based on a large cohort of elderly dialysis patients, enrolling 377 patients with long-term follow-up (2012–2024), featuring high data completeness. This allows for a comprehensive reflection of the impact of different dialysis modalities (HD vs. PD) on the prognosis of elderly patients, particularly capturing the cumulative effects of long-term complications. Secondly, through detailed baseline status analysis and multivariate Cox regression models, the study deeply explored key factors influencing prognosis, precisely identifying independent risk factors and protective factors associated with survival. Furthermore, utilizing the competing risk model (cmprsk) to compare causes of death in detail across different dialysis modalities revealed the significant impact of hemorrhagic diseases in hemodialysis. Combining Kaplan-Meier survival curves and Log-Rank tests, the study visually demonstrated significant differences in long-term survival rates between dialysis modalities, making the results more accessible for clinicians and patients to understand. Finally, the study conducted meticulous classification and in-depth analysis of causes of death, providing an important basis for improving treatment strategies for elderly dialysis patients.

However, this study also has several limitations.First, a substantial number of deaths lacked a clearly identified cause during investigation, which may lead to an underestimation of the true incidence of various causes of mortality.Second, the investigation of hemorrhagic diseases did not consider patients’ use of anticoagulants and antiplatelet agents, which may significantly influence bleeding risk. Furthermore, the study only analyzed fatal bleeding events, while a large number of non-fatal bleeding episodes were not included, potentially underestimating the overall burden of bleeding complications. Regarding dialysis prescription details, this study did not systematically collect or analyze hemodialysis frequency, dialysis dose (e.g., Kt/V), or specific treatment modalities (such as online hemodiafiltration [HDF] or hemoperfusion [HP]). For peritoneal dialysis, no distinction was made between continuous ambulatory peritoneal dialysis (CAPD) and automated peritoneal dialysis (APD), nor were details on dialysate type (e.g., glucose concentration, icodextrin use) recorded. Consequently, we cannot assess whether differences in outcomes are attributable to the dialysis modality per se or to variations in treatment adequacy within each modality. Differences in these treatment modalities and prescription specifics may directly affect solute clearance, volume control, hemodynamic stability, and metabolic outcomes, thereby influencing the precise interpretation of between-group prognosis comparisons.In terms of nutritional assessment, this study primarily relied on laboratory indicators (e.g., albumin, prealbumin) and lacked a more comprehensive clinical nutritional evaluation, which may limit a complete understanding of the impact of nutritional status on prognosis. Moreover, due to the limited number of specific events, many risk factor analyses in this study—particularly within comorbidity subgroups—yielded hazard ratios (HRs) with wide confidence intervals, and a large proportion of results did not reach statistical significance. These extreme or borderline findings should not be considered conclusive and require validation through future studies with larger sample sizes; therefore, caution is warranted when applying these results in clinical practice. The study also suffered from insufficient assessment of residual renal function at dialysis initiation, especially the difficulty in accurately evaluating it in hemodialysis patients, which may affect a comprehensive understanding of the relationship between dialysis modality and prognosis. Missing data existed for some variables in the dataset, which may have influenced the accuracy of the analytical results to some extent. Regarding statistical methodology, the competing risk model had a limited ability to adjust for potential confounders, including socioeconomic and healthcare-access factors, and treated laboratory covariates as time-fixed, without accounting for their longitudinal variation during follow-up, which may influence risk estimates. Finally, as a single-center study, the findings may be subject to selection bias, potentially influenced by local clinical practices, patient preferences, and shared decision-making. Therefore, the generalizability of our conclusions requires confirmation in multi-center studies.

## Conclusions and recommendations

### Conclusions

This study supports that dialysis modality selection for elderly ESRD patients requires individualized decision-making based on comorbidity profiles. Our findings indicate that hemodialysis may be associated with a survival benefit in the overall population and appears to be a particularly relevant strategy for patients with concomitant heart failure or diabetes. Conversely, peritoneal dialysis may be more beneficial for patients with cerebrovascular disease who are at higher risk of bleeding. Metabolic management and cardiac function monitoring are crucial for prognosis. However, given the inherent limitations of a retrospective, single-center design, these observations warrant validation through large-scale, prospective studies to confirm the causal inferences and generalizability of these relationships.

**Fig. 3:**
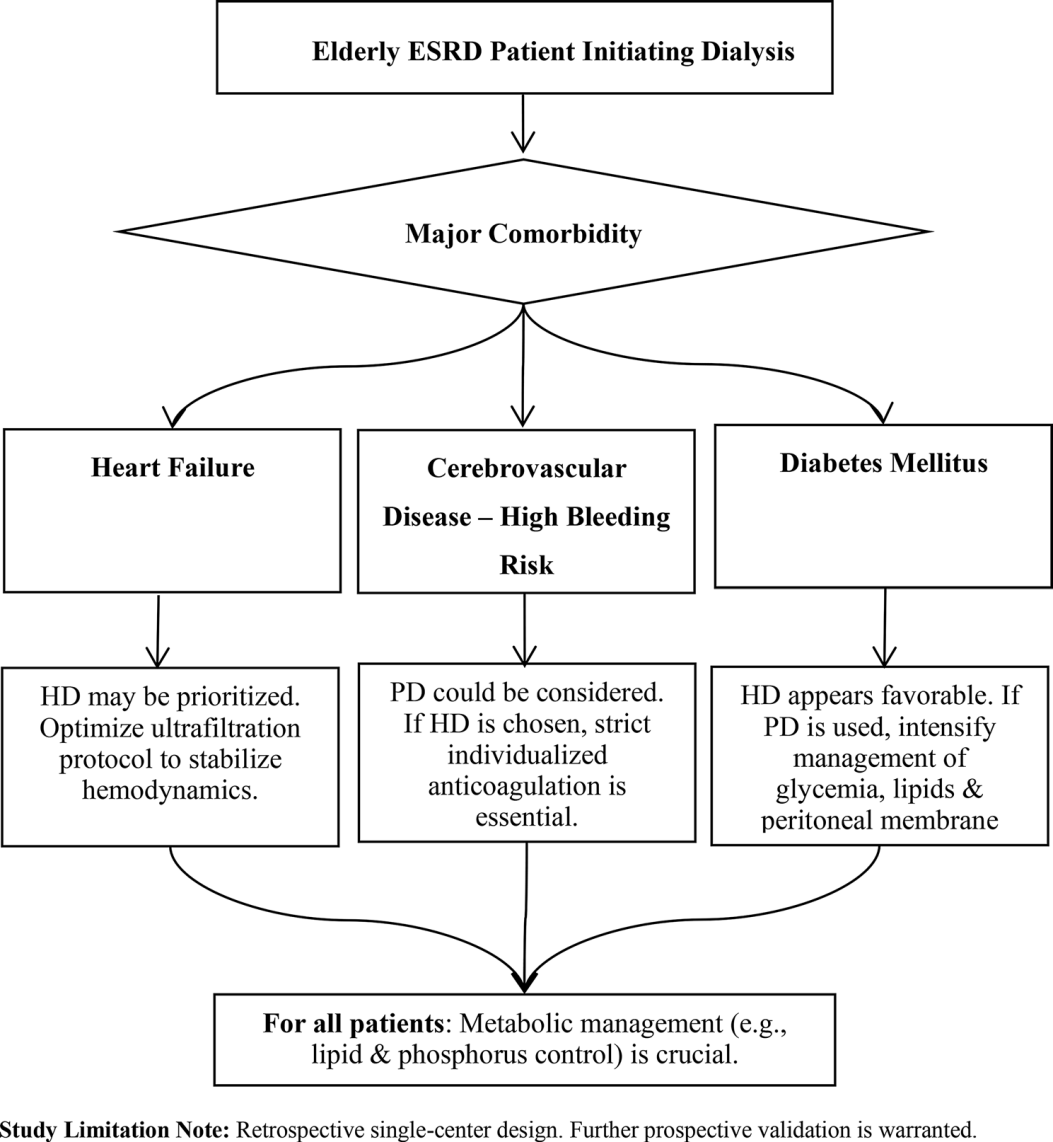
A comorbidity-guided approach to dialysis modality selection in elderly ESRD patients

### Recommendations

In clinical practice, adopting a “comorbidity-oriented” individualized dialysis strategy is recommended (Fig. 3): in patients with heart failure, HD might be prioritised, with care taken to optimise ultrafiltration protocols to stabilise haemodynamics.For those with cerebrovascular disease at high risk of bleeding, PD could be considered; if HD is necessary, a strict, individualised anticoagulation regimen is essential.For diabetic patients, HD appears favourable; if PD is used, intensified management of glycaemia, lipids, and peritoneal membrane function is warranted. Future research should further conduct multi-center studies integrating residual renal function and novel biomarkers to refine risk stratification models and optimize individualized treatment plans for dialysis patients.

## Supplementary Information

Below is the link to the electronic supplementary material.

Supplementary Material 1

Supplementary Material 2

## Data Availability

Data is provided within the manuscript or supplementary information files.

## References

[1] Chen Z, Yu C. The current status and trends of china’s elderly population and disease burden from a global perspective. J Public Health Prev Med. 2025;36(03):1–6.

[2] Zhou L, Zeng XX, Fu P. Community Hemodialysis in china: opportunities and challenges. Chin Med J (Engl). 2017;130(18):2143–6. 28875949 10.4103/0366-6999.213961 PMC5598324

[3] Segall L, Nistor I, Van Biesen W, Brown EA, Heaf JG, Lindley E, Farrington K, Covic A. Dialysis modality choice in elderly patients with end-stage renal disease: a narrative review of the available evidence. Nephrol Dial Transpl. 2017;32(1):41–9. 10.1093/ndt/gfv411 26673908

[4] Cheng L, Hu N, Song D, Chen Y. Mortality of peritoneal Dialysis versus Hemodialysis in older adults: an updated systematic review and meta-analysis. Gerontology. 2024;70(5):461–78. 38325351 10.1159/000536648 PMC11098023

[5] Ahmed FA, Catic AG. Decision-making in geriatric patients with end-stage renal disease: thinking beyond nephrology. J Clin Med 2018, 8(1). 10.3390/jcm8010005 PMC6352025 30577486

[6] Guo L, Ji Y, Sun T, Liu Y, Jiang C, Wang G, Xing H, Yang B, Xu A, Xian X, et al. Management of chronic heart failure in Dialysis patients: A challenging but rewarding path. Rev Cardiovasc Med. 2024;25(6):232. 39076321 10.31083/j.rcm2506232 PMC11270084

[7] Chen TH, Wen YH, Chen CF, Tan AC, Chen YT, Chen FY, Lin CC. The advantages of peritoneal Dialysis over Hemodialysis during the COVID-19 pandemic. Semin Dial. 2020;33(5):369–71. 32677120 10.1111/sdi.12903 PMC7405464

[8] He Z, Liang H, Huang J, Zhang D, Ma H, Lin J, Cai Y, Liu T, Li H, Qiu W, et al. Impact of Dialysis modality choice on the survival of end-stage renal disease patients with congestive heart failure in Southern china: A retrospective cohort study. Front Med (Lausanne). 2022;9:898650. 36330070 10.3389/fmed.2022.898650 PMC9623394

[9] Zhao Y. Comparison of the effect of Hemodialysis and peritoneal Dialysis in the treatment of end-stage renal disease. Pak J Med Sci. 2023;39(6):1562–7. 37936738 10.12669/pjms.39.6.8056 PMC10626077

[10] Zhang P, Xun L, Bao N, Tong D, Duan B, Peng D. Long-term mortality in patients with end-stage renal disease undergoing Hemodialysis and peritoneal dialysis: a propensity score matching retrospective study. Ren Fail. 2024;46(1):2321320. 38482569 10.1080/0886022X.2024.2321320 PMC10946263

[11] Yang F, Khin LW, Lau T, Chua HR, Vathsala A, Lee E, Luo N. Hemodialysis versus peritoneal dialysis: A comparison of survival outcomes in south-east Asian patients with end-stage renal disease. PLoS ONE. 2015;10(10):e0140195. 26444003 10.1371/journal.pone.0140195 PMC4622046

[12] Foley RN, Parfrey PS, Harnett JD, Kent GM, O’Dea R, Murray DC, Barre PE. Mode of Dialysis therapy and mortality in end-stage renal disease. J Am Soc Nephrol. 1998;9(2):267–76. 9527403 10.1681/ASN.V92267

[13] McDonald SP, Marshall MR, Johnson DW, Polkinghorne KR. Relationship between Dialysis modality and mortality. J Am Soc Nephrol. 2009;20(1):155–63. 19092128 10.1681/ASN.2007111188 PMC2615722

[14] Lukowsky LR, Mehrotra R, Kheifets L, Arah OA, Nissenson AR, Kalantar-Zadeh K. Comparing mortality of peritoneal and Hemodialysis patients in the first 2 years of Dialysis therapy: a marginal structural model analysis. Clin J Am Soc Nephrol. 2013;8(4):619–28. 23307879 10.2215/CJN.04810512 PMC3613949

[15] Kumar VA, Sidell MA, Jones JP, Vonesh EF. Survival of propensity matched incident peritoneal and Hemodialysis patients in a united States health care system. Kidney Int. 2014;86(5):1016–22. 24988066 10.1038/ki.2014.224

[16] Banshodani M, Kawanishi H, Moriishi M, Shintaku S, Tsuchiya S. Association between Dialysis modality and cardiovascular diseases: A comparison between peritoneal Dialysis and Hemodialysis. Blood Purif. 2020;49(3):302–9. 31851981 10.1159/000504040

[17] Weinhandl ED, Foley RN, Gilbertson DT, Arneson TJ, Snyder JJ, Collins AJ. Propensity-matched mortality comparison of incident Hemodialysis and peritoneal Dialysis patients. J Am Soc Nephrol. 2010;21(3):499–506. 20133483 10.1681/ASN.2009060635 PMC2831857

[18] Peng YK, Tai TS, Wu CY, Tsai CY, Lee CC, Chen JJ, Hsiao CC, Chen YC, Yang HY, Yen CL. Clinical outcomes between elderly ESKD patients under peritoneal Dialysis and hemodialysis: a National cohort study. Sci Rep. 2023;13(1):16199. 37758848 10.1038/s41598-023-43476-1 PMC10533893

[19] Sens F, Schott-Pethelaz AM, Labeeuw M, Colin C, Villar E. Survival advantage of Hemodialysis relative to peritoneal Dialysis in patients with end-stage renal disease and congestive heart failure. Kidney Int. 2011;80(9):970–7. 21775972 10.1038/ki.2011.233

[20] Khan U, Siddiqui MA. Comparative analysis of peritoneal Dialysis and Hemodialysis for the management of kidney failure: A concise review. J Pak Med Assoc. 2024;74(3):617. 38591321 10.47391/JPMA.10296

[21] van Eck A, Bonenkamp AA, van Wallene VA, Hoekstra T, Lissenberg-Witte BI, Dekker FW, van Ittersum FJ, Verhaar MC, van Jaarsveld BC, Abrahams AC. Differences in hospitalisation between peritoneal Dialysis and haemodialysis patients. Eur J Clin Invest. 2022;52(6):e13758. 35129213 10.1111/eci.13758 PMC9286659

[22] Lu JC, Lee P, Ierino F, MacIsaac RJ, Ekinci E, O’Neal D. Challenges of glycemic control in people with diabetes and advanced kidney disease and the potential of automated insulin delivery. J Diabetes Sci Technol. 2024;18(6):1500–8. 37162092 10.1177/19322968231174040 PMC11531035

[23] Bronas UG, Puzantian H, Hannan M. Cognitive impairment in chronic kidney disease: vascular milieu and the potential therapeutic role of exercise. Biomed Res Int 2017, 2017:2726369. 10.1155/2017/2726369 PMC5414492 28503567

[24] Kelly D, Rothwell PM. Disentangling the multiple links between renal dysfunction and cerebrovascular disease. J Neurol Neurosurg Psychiatry. 2020;91(1):88–97. 31511306 10.1136/jnnp-2019-320526 PMC6952845

[25] Iseki K, Fukiyama K. Clinical demographics and long-term prognosis after stroke in patients on chronic haemodialysis. The Okinawa Dialysis study (OKIDS) group. Nephrol Dial Transpl. 2000;15(11):1808–13. 10.1093/ndt/15.11.1808 11071969

[26] Belmar L, de Francisco AL, Bueno L, Piñera C, Monfá E, Kislikova M, Seras M, Rodrigo E, Arias M. Strokes in patients on haemodialysis: incidence, onset time and associated factors. Nefrologia. 2014;34(3):347–52. 24849056 10.3265/Nefrologia.pre2014.Jan.12298

[27] Murakami M, Hamasaki T, Kimura S, Maruyama D, Kakita K. Clinical features and management of intracranial hemorrhage in patients undergoing maintenance Dialysis therapy. Neurol Med Chir (Tokyo). 2004;44(5):225–32. discussion 233. 15200056 10.2176/nmc.44.225

[28] Miglinas M, Cesniene U, Janusaite MM, Vinikovas A. Cerebrovascular disease and cognition in chronic kidney disease patients. Front Cardiovasc Med. 2020;7:96. 32582768 10.3389/fcvm.2020.00096 PMC7283453

[29] Cheng M, Ding Y, Kim E, Geng X. Exploring the therapeutic potential of peritoneal Dialysis (pd) in the treatment of neurological disorders. Cell Transpl. 2024;33:9636897241236576. 10.1177/09636897241236576 PMC10956140 38506429

[30] Barbu E, Mihaila AC, Gan AM, Ciortan L, Macarie RD, Tucureanu MM, Filippi A, Stoenescu AI, Petrea SV, Simionescu M et al. The elevated inflammatory status of neutrophils is related to in-hospital complications in patients with acute coronary syndrome and has important prognosis value for diabetic patients. Int J Mol Sci 2024, 25(10). 10.3390/ijms25105107 PMC11121518 38791147

[31] Pang L, Ding Z, Chai H, Shuang W. The causal relationship between immune cells and different kidney diseases: A Mendelian randomization study. Open Med (Wars). 2023;18(1):20230877. 38152332 10.1515/med-2023-0877 PMC10751893

[32] Herrero-Cervera A, Soehnlein O, Kenne E. Neutrophils in chronic inflammatory diseases. Cell Mol Immunol. 2022;19(2):177–91. 35039631 10.1038/s41423-021-00832-3 PMC8803838

[33] Liu H, He Z, Hu X, Li S, Wang L, Zhao D, Lin Q, Liu X, Lu F, Zhang D. Propensity-matched comparison of mortality between peritoneal Dialysis and Hemodialysis in patients with type 2 diabetes. Int Urol Nephrol. 2022;54(6):1373–81. 34657242 10.1007/s11255-021-03026-y

[34] Krediet RT. Long-term peritoneal dialysis-impact of peritoneal membrane changes and newer solutions. Int J Artif Organs. 1998;21(8):437–9. 9803343

[35] Xu XD, Han X, Yang Y, Li X. Comparative study on the efficacy of peritoneal Dialysis and Hemodialysis in patients with end-stage diabetic nephropathy. Pak J Med Sci. 2020;36(7):1484–9. 33235561 10.12669/pjms.36.7.2901 PMC7674865

[36] Shen Y. Pathogenesis and mechanism of uremic vascular calcification. Cureus. 2024;16(7):e64771. 39026575 10.7759/cureus.64771 PMC11255132

[37] de Moraes TP, Fortes PC, Ribeiro SC, Riella MC, Pecoits-Filho R. Comparative analysis of lipid and glucose metabolism biomarkers in non-diabetic Hemodialysis and peritoneal Dialysis patients. J Bras Nefrol. 2011;33(2):173–9. 21789431 10.1590/s0101-28002011000200009

