# Supplementary material for: Hemodialysis versus peritoneal dialysis in elderly ESRD patients: a retrospective cohort study on survival and the role of comorbidities
Source: BMC Nephrol. 2026 Jan 30;27:142. doi: 10.1186/s12882-026-04783-7 (PMC12930720; doi:10.1186/s12882-026-04783-7)
Supplement: Supplementary file 1 — Supplementary Material 1 [file 12882_2026_4783_MOESM1_ESM.pdf]

SUPPLEMENTARY MATERIAL

Hemodialysis versus peritoneal dialysis in elderly ESRD patients: A retrospective cohort study on survival and the role of comorbidities

TABLE OF CONTENTS

Supplementary Table S1: Comparison of the cumulative incidence of cardiovascular disease deaths in the hemodialysis and peritoneal dialysis groups.

Supplementary Table S2: Comparison of the cumulative incidence of death from cerebrovascular disease in the hemodialysis and peritoneal dialysis groups.

Supplementary Table S3: Variance Inflation Factor (VIF) for Variables in Multivariable Cox Regression Models

Supplementary Table S4: Univariable competing risk regression for the overall population analyzing risk factors for cerebrovascular disease.

Supplementary Table S5: Univariate to multivariate competing risk Cox regression models for the overall population analyzing risk factors for cardiovascular disease mortality.

Supplementary Table S6: Univariate to multivariate Cox regression models analyzing risk factors for all-cause mortality in a heart failure population.

Supplementary Table S7: Univariate to multivariate competing risk Cox regression model analysis of risk factors for hemorrhagic death in a population with comorbid heart failure disease.

Supplementary Table S8: Univariate to multivariate competing risk Cox regression model analysis of risk factors for cerebrovascular disease death in a population with comorbid heart failure disease.

Supplementary Table S9: Univariate to multivariate competing risk Cox regression model analysis of risk factors for cardiovascular disease death in a population with comorbid heart failure disease.

Supplementary Table S10: Univariate to multivariate Cox regression model analysis of risk factors for all-cause mortality in a population with combined cerebrovascular disease.

Supplementary Table S11: Univariate to multivariate competing risk Cox regression model analysis of risk factors for hemorrhagic death in a population with comorbid cerebrovascular disease.

Supplementary Table S12: Univariate to multivariate competing risk Cox regression model analysis of risk factors for cerebrovascular disease death in a population with comorbid cerebrovascular disease.

Supplementary Table S13: Univariate to multivariate competing risk Cox regression model analysis of risk factors for cardiovascular disease death in a population with comorbid cerebrovascular disease.

Supplementary Table S14: Univariate to multivariate Cox regression models analyzing risk factors for all-cause mortality in the comorbid diabetic population.

Supplementary Table S15: Univariate to multivariate competing risk Cox regression models analyzing risk factors for hemorrhagic death in the comorbid diabetes population.

Supplementary Table S16: Univariate to multivariate competing risk Cox regression models analyzing risk factors for cerebrovascular death in the comorbid diabetes population.

Supplementary Table S17: Univariate to multivariate competing risk Cox regression models analyzing risk factors for cardiovascular disease death in the comorbid diabetes population.

Supplementary Figure S1: Cumulative incidence of all-cause mortality in the overall population and comparison with that in hemodialysis and peritoneal dialysis groups.

Supplementary Figure S2: Cumulative incidence of death from infectious disease in the overall population and comparison with that in hemodialysis and peritoneal dialysis groups.

Supplementary Figure S3: Cumulative incidence of death from cardiovascular disease in the overall population and comparison with that in hemodialysis and peritoneal dialysis groups.

Supplementary Figure S4: Cumulative incidence of death from cerebrovascular disease in the overall population and comparison with that in hemodialysis and peritoneal dialysis groups.

Supplementary Figure S5: Cumulative incidence of death from hemorrhagic disease in the overall population and comparison with that in hemodialysis and peritoneal dialysis groups.

Supplementary Figure S6: Cumulative incidence of death from other disease in the overall population and comparison with that in hemodialysis and peritoneal dialysis groups.

Supplementary Figure S7: Survival comparison between groups with and without heart failure in the overall patients, hemodialysis patients, and peritoneal dialysis patients.

Supplementary Figure S8: Survival comparison between groups with and without cerebrovascular disease in the overall patients, hemodialysis patients, and peritoneal dialysis patients.

Supplementary Figure S9: Survival comparison between groups with and without diabetes mellitus in the overall patients, hemodialysis patients, and peritoneal dialysis patients.

Table S1: Comparison of the cumulative incidence of cardiovascular disease deaths in the hemodialysis and peritoneal dialysis groups.

	**Overall situation**	**HD group**	**PD group**
Incident rate	42.97%	42.49%	43.53%
Cumulative 12-month incidence rate	1.11%	0.96%	1.31%
Cumulative 24-month incidence rate	5.59%	5.81%	5.15%
Cumulative 60-month incidence rate	19.57%	18.65%	21.51%
Cumulative 120-month incidence rate	42.97%	42.49%	43.53%

HD, hemodialysis;PD, peritoneal dialysis.

Table S2: Comparison of the cumulative incidence of death from cerebrovascular disease in the hemodialysis and peritoneal dialysis groups.

	**Overall situation**	**HD group**	**PD group**
Incident rate	17.75%	18.10%	14.86%
Cumulative 12-month incidence rate	0.28%	0.49%	0.00%
Cumulative 24-month incidence rate	0.88%	1.50%	0.00%
Cumulative 60-month incidence rate	4.37%	4.63%	4.22%
Cumulative 120-month incidence rate	17.75%	18.10%	14.86%

HD, hemodialysis;PD, peritoneal dialysis.

Table S3: Variance Inflation Factor (VIF) for Variables in Multivariable Cox Regression Models

**Variable**	**VIF**
VIF for Model 2	
sex	1.018356
dialysis initiation age	1.018356
VIF for Model 3	
sex	1.01963
dialysis initiation age	1.040275
coronary artery disease	1.021774
VIF for Model 4	
sex	1.073514
dialysis initiation age	1.082858
CCI without age	1.084121
serum Plasma albumin	1.081597

HDLC	1.056764
serum phosphorus	1.072011
VIF for Model 5	
sex	1.07522
dialysis initiation age	1.083142
CCI without age	1.096244
serum Plasma albumin	1.081821
HDLC	1.057104
serum phosphorus	1.072338
left ventricular ejection fraction	1.01804

HDLC, high-density lipoprotein cholesterol.

Table S4: Univariable competing risk regression for the overall population analyzing risk factors for cerebrovascular disease.

	**Model1 HR [95%CI]**
Dialysis type(PD vs. HD)	1.832[0.58-5.75]
Sex(female vs. male)	0.397[0.13-1.23]
Age at dialysis initiation(per 1 year)	0.955[0.88-1.04]
BMI(per 1kg/m2)	0.951[0.87-1.03]
Diabetes mellitus(Yes vs. No)	0.684[0.24-1.92]
CAD heart(Yes vs. No)	0.724[0.21-2.52]
CCI without age(per 1 point)	0.894[0.68-1.17]
EF(per 1%)	1.004[0.96-1.05]
FS(per 1%)	1.020[0.96-1.09]
Hemoglobin(per 1g/L)	0.969[0.94-1.0]
Plasma albumin(per 1g/L)	1.002[0.94-1.07]
Total cholesterol(per 1 mmol/L)	1.002[0.8-1.25]
Triglyceride(per 1 mmol/L)	0.907[0.75-1.1]
HDLC(per 1 mmol/L)	0.239[0.04-1.27]
LDLC(per 1 mmol/L)	1.086[0.86-1.37]
UA(per 1 μmol/L)	1.003[1.0-1.01]
Phosphorus(per 1 mmol/L)	1.048[0.32-3.46]
Calcium(per 1 mmol/L)	0.400[0.04-3.94]

HR, hazard ratio; 95% CI, 95% confidence interval; HD, hemodialysis; PD, peritoneal dialysis; BMI, body mass index; CAD, coronary artery disease; CCI, Charlson Comorbidity Index (calculated without age component); EF, left ventricular ejection fraction; FS, left ventricular fractional shortening;HDLC, high-density lipoprotein cholesterol; LDLC, low-density lipoprotein cholesterol;UA, uric acid; Model 1: Univariate analysis.

Table S5: Univariate to multivariate competing risk Cox regression models for the overall population analyzing risk factors for cardiovascular disease mortality.

	**Model1 HR [95%CI]**	**Model2 HR [95%CI]**	**Model3 HR [95%CI]**	**Model4 HR [95%CI]**	**Model5 HR [95%CI]**
Dialysis type(PD vs. HD)	1.093[0.69-1.73]	1.015[0.64-1.61]	0.950[0.6-1.5]	0.923[0.58-1.46]	0.992[0.63-1.57]
Sex(female vs. male)	0.951[0.61-1.48]	0.901[0.58-1.4]	0.908[0.59-1.41]	0.943[0.6-1.47]	0.959[0.61-1.51]
Age at dialysis initiation(per 1 year)	1.036[1.0-1.07]	1.036[1.0-1.07]	1.034[1.0-1.07]	1.033[1.0-1.07]	1.033[1.0-1.07]

BMI(per 1kg/m2)	1.046[1.01-1.08]	1.049[1.02-1.08]	1.033[1.01-1.06]	1.022[0.99-1.05]	1.011[0.98-1.04]
Diabetes mellitus(Yes vs. No)	2.272[1.42-3.63]	2.271[1.42-3.64]	2.129[1.32-3.42]	1.845[0.98-3.47]	1.931[1.19-3.14]
CAD heart(Yes vs. No)	2.311[1.48-3.62]	2.161[1.36-3.44]	2.077[1.27-3.38]	1.847[1.13-3.01]	1.804[1.1-2.97]
CCI without age(per 1 point)	1.265[1.12-1.43]	1.254[1.1-1.42]	1.207[1.05-1.38]	1.207[1.05-1.38]	1.051[0.87-1.27]
EF(per 1%)	0.966[0.95-0.98]	0.965[0.95-0.98]	1.014[0.96-1.07]	1.010[0.96-1.06]	1.025[0.97-1.08]
FS(per 1%)	0.943[0.91-0.97]	0.944[0.91-0.97]	0.922[0.84-1.01]	0.932[0.86-1.02]	0.917[0.84-1.0]
Hemoglobin(per 1g/L)	1.017[1.01-1.03]	1.015[1.01-1.03]	1.017[1.01-1.03]	1.017[1.01-1.03]	1.018[1.01-1.03]
Plasma albumin(per 1g/L)	1.016[0.97-1.06]	1.011[0.97-1.06]	0.994[0.95-1.04]	1.003[0.96-1.05]	1.002[0.96-1.05]
Total cholesterol(per 1 mmol/L)	1.097[0.9-1.33]	1.125[0.92-1.37]	1.127[0.93-1.37]	1.13[0.93-1.37]	1.106[0.92-1.33]
Triglyceride(per 1 mmol/L)	1.102[0.97-1.26]	1.122[0.99-1.28]	1.126[0.99-1.28]	1.105[0.97-1.26]	1.073[0.94-1.23]
HDLC(per 1 mmol/L)	0.907[0.41-1.99]	0.856[0.39-1.86]	0.772[0.37-1.63]	0.881[0.41-1.88]	0.908[0.43-1.91]
LDLC(per 1 mmol/L)	1.126[0.89-1.43]	1.167[0.92-1.48]	1.174[0.94-1.47]	1.189[0.95-1.49]	1.171[0.95-1.45]
UA(per 1 μmol/L)	1.000[1.0-1.0]	1.000[1.0-1.0]	1.001[1.0-1.0]	1.001[1.0-1.0]	1.001[1.0-1.0]
Phosphorus(per 1 mmol/L)	0.971[0.65-1.46]	1.062[0.71-1.59]	1.080[0.72-1.62]	1.152[0.77-1.72]	1.191[0.78-1.82]
Calcium(per 1 mmol/L)	1.574[0.63-3.92]	1.353[0.55-3.36]	1.008[0.42-2.42]	1.000[0.41-2.43]	1.014[0.43-2.38]

HR, hazard ratio; 95% CI, 95% confidence interval; HD, hemodialysis; PD, peritoneal dialysis; BMI, body mass index; CAD, coronary artery disease; CCI, Charlson Comorbidity Index (calculated without age component); EF, left ventricular ejection fraction; FS, left ventricular fractional shortening;HDLC, high-density lipoprotein cholesterol; LDLC, low-density lipoprotein cholesterol;UA, uric acid; Model 1: Univariate analysis; Model 2: Multivariate analysis adjusted for Age at dialysis initiation; Model 3: Multivariate analysis adjusted for Age at dialysis initiation, BMI, EF, FS and Hemoglobin; Model 4: Multivariate analysis adjusted for Age at dialysis initiation, CCI without age, BMI, EF, FS and Hemoglobin; Model 5: Multivariate analysis adjusted for Age at dialysis initiation, DM, CAD, BMI, EF, FS and Hemoglobin.

Table S6: Univariate to multivariate Cox regression models analyzing risk factors for all-cause mortality in a heart failure population.

	**Model1 HR [95%CI]**	**Model2 HR [95%CI]**	**Model3 HR [95%CI]**	**Model4 HR [95%CI]**	**Model5 HR [95%CI]**
Dialysis type(PD vs. HD)	0.529[0.33-0.84]	0.436[0.27-0.71]	0.442[0.27-0.73]	0.422[0.25-0.7]	0.456[0.26-0.79]
Sex(female vs. male)	0.685[0.44-1.06]	0.571[0.36-0.9]	0.553[0.35-0.88]	0.529[0.33-0.84]	0.554[0.35-0.88]
Age at dialysis itiation(per 1 year)	1.042[1.01-1.08]	1.042[1.01-1.08]	1.036[1.0-1.07]	1.035[1.0-1.07]	1.053[1.02-1.09]
BMI(per 1kg/m2)	0.977[0.93-1.02]	0.976[0.93-1.02]	0.978[0.93-1.02]	0.976[0.93-1.02]	0.982[0.94-1.02]
HBP(per 1mmHg)	3.569[0.5-25.72]	2.833[0.39-20.62]	2.915[0.4-21.29]	3.172[0.43-23.2]	3.142[0.43-22.86]
CAD(Yes vs. No)	1.630[1.04-2.55]	1.467[0.93-2.32]	1.467[0.93-2.32]	1.456[0.92-2.3]	1.305[0.8-2.14]
CCI without age(per 1 point)	1.089[0.93-1.27]	1.138[0.97-1.33]	1.124[0.96-1.32]	1.127[0.96-1.32]	1.124[0.95-1.33]
EF(per 1%)	0.983[0.97-1.0]	0.978[0.96-1.0]	0.981[0.96-1.0]	0.979[0.96-1.0]	0.982[0.96-1.0]
FS(per 1%)	0.975[0.95-1.0]	0.967[0.94-1.0]	0.972[0.94-1.0]	0.971[0.94-1.0]	1.014[0.91-1.13]
Hemoglobin(per 1g/L)	0.998[0.99-1.01]	0.996[0.99-1.01]	0.996[0.99-1.01]	0.996[0.99-1.01]	0.999[0.99-1.01]
Plasma albumin(per 1g/L)	0.998[0.96-1.04]	0.980[0.94-1.03]	0.982[0.94-1.03]	0.986[0.94-1.03]	0.988[0.94-1.04]
Total cholesterol(per 1 mmol/L)	1.176[0.95-1.46]	1.158[0.92-1.45]	1.163[0.93-1.45]	0.766[0.43-1.38]	0.982[0.52-1.84]
Triglyceride(per 1 mmol/L)	1.050[0.91-1.21]	1.093[0.94-1.27]	1.097[0.94-1.28]	1.105[0.93-1.32]	1.170[0.99-1.39]
HDLC(per 1 mmol/L)	0.677[0.3-1.53]	0.521[0.22-1.23]	0.569[0.24-1.37]	0.489[0.19-1.23]	0.561[0.22-1.44]
LDLC(per 1 mmol/L)	1.283[1.01-1.63]	1.280[1.0-1.64]	1.274[0.99-1.63]	1.274[0.99-1.63]	1.304[1.03-1.66]
UA(per 1 μmol/L)	1.000[1.0-1.0]	1.000[1.0-1.0]	1.001[1.0-1.0]	1.001[1.0-1.0]	1.001[1.0-1.0]
Phosphorus(per 1 mmol/L)	0.730[0.47-1.14]	0.803[0.51-1.26]	0.841[0.53-1.33]	0.854[0.54-1.36]	1.048[0.66-1.67]
Calcium(per 1 mmol/L)	1.234[0.58-2.63]	1.094[0.5-2.38]	1.140[0.52-2.49]	1.246[0.57-2.71]	1.117[0.51-2.47]

HR, hazard ratio; 95%CI, 95% confidence interval; HD, hemodialysis; PD, peritoneal dialysis; BMI, body mass index; HBP, hypertension; CAD, coronary artery disease; CCI, Charlson Comorbidity Index (calculated without age component); EF, left ventricular ejection fraction; FS, left

ventricular fractional shortening; HDLC, high-density lipoprotein cholesterol; LDLC, low-density lipoprotein cholesterol; UA, uric acid; *Model 1*: Univariate analysis;*Model 2*: Multivariate analysis adjusted for dialysis initiation age; *Model 3*: Multivariate analysis adjusted for dialysis initiation age and CAD; *Model 4*: Multivariate analysis adjusted for dialysis initiation age, CAD and LDLC; *Model 5*: Multivariate analysis adjusted for dialysis initiation age, CAD, LDLC, EF and sex.

Table S7: Univariate to multivariate competing risk Cox regression model analysis of risk factors for hemorrhagic death in a population with comorbid heart failure disease.

	**Model1 HR [95%CI]**	**Model2 HR [95%CI]**
Dialysis type(PD vs. HD)	2.758[0.33-23.3]	1.845[0.23-14.49]
Sex(female vs. male)	0.908[0.19-4.43]	1.778[0.46-6.82]
Age at dialysis initiation(per 1 year)	0.976[0.9-1.06]	1.004[0.91-1.11]
BMI(per 1kg/m2)	0.933[0.71-1.23]	0.932[0.66-1.31]
Diabetes mellitus(Yes vs. No)	0.572[0.12-2.74]	0.501[0.11-2.36]
CAD heart(Yes vs. No)	0.989[0.21-4.73]	1.848[0.4-8.63]
CCI without age(per 1 point)	0.807[0.48-1.37]	0.860[0.37-2.02]
EF(per 1%)	0.998[0.94-1.06]	0.998[0.94-1.06]
FS(per 1%)	1.034[0.94-1.13]	1.031[0.95-1.12]
Hemoglobin(per 1g/L)	0.972[0.93-1.01]	0.976[0.93-1.02]
Plasma albumin(per 1g/L)	1.003[0.91-1.11]	1.039[0.93-1.16]
Total cholesterol(per 1 mmol/L)	1.087[0.8-1.47]	1.110[0.79-1.55]
Triglyceride(per 1 mmol/L)	1.007[0.82-1.23]	1.113[0.9-1.38]
HDLC(per 1 mmol/L)	0.067[0.0-1.36]	0.104[0.0-3.94]
LDLC(per 1 mmol/L)	1.099[0.85-1.41]	1.089[0.77-1.55]
UA(per 1 μmol/L)	1.000[1.0-1.0]	1.000[1.0-1.0]
Phosphorus(per 1 mmol/L)	0.971[0.65-1.46]	1.062[0.71-1.59]
Calcium(per 1 mmol/L)	1.574[0.63-3.92]	1.353[0.55-3.36]

HR, hazard ratio; 95% CI, 95% confidence interval; HD, hemodialysis; PD, peritoneal dialysis; BMI, body mass index; CAD, coronary artery disease; CCI, Charlson Comorbidity Index (calculated without age component); EF, left ventricular ejection fraction; FS, left ventricular fractional shortening;HDLC, high-density lipoprotein cholesterol; LDLC, low-density lipoprotein cholesterol;UA, uric acid; Model 1: Univariate analysis;Model 2: Multivariate analysis adjusted for UA.

Table S8: Univariate to multivariate competing risk Cox regression model analysis of risk factors for cerebrovascular disease death in a population with comorbid heart failure disease.

	**Model1 HR [95%CI]**	**Model2 HR [95%CI]**	**Model3 HR [95%CI]**
Dialysis type(PD vs. HD)	0.562[0.08-4.09]	0.555[0.09-3.61]	0.757[0.09-6.52]
Sex(female vs. male)	0.302[0.03-2.82]	0.389[0.04-3.61]	0.610[0.04-9.31]
Age at dialysis initiation(per 1 year)	0.840[0.72-0.98]	0.840[0.72-0.98]	0.900[0.82-0.99]
BMI(per 1kg/m2)	0.927[0.84-1.03]	0.923[0.83-1.03]	1.059[0.96-1.17]
Diabetes mellitus(Yes vs. No)	0.554[0.08-3.87]	0.448[0.07-3.04]	7.720[0.11-560.23]
CAD heart(Yes vs. No)	1.013[0.15-6.76]	1.17[0.19-7.18]	1.447[0.17-12.52]
CCI without age(per 1 point)	0.715[0.32-1.58]	0.692[0.36-1.34]	1.084[0.64-1.85]

EF(per 1%)	0.969[0.92-1.02]	0.981[0.93-1.04]	1.006[0.89-1.14]
FS(per 1%)	0.991[0.93-1.06]	1.013[0.95-1.07]	1.152[0.99-1.34]
Hemoglobin(per 1g/L)	0.968[0.92-1.02]	0.957[0.89-1.03]	0.957[0.87-1.05]
Plasma albumin(per 1g/L)	0.933[0.85-1.03]	0.971[0.87-1.08]	1.058[0.93-1.2]
Total cholesterol(per 1 mmol/L)	1.065[0.71-1.61]	1.040[0.7-1.54]	0.617[0.29-1.32]
Triglyceride(per 1 mmol/L)	0.991[0.77-1.28]	0.888[0.68-1.15]	1.078[0.78-1.5]
HDLC(per 1 mmol/L)	0.073[0.0-22.82]	0.117[0.0-14.54]	0.001[0.0-0.21]
LDLC(per 1 mmol/L)	1.061[0.78-1.45]	1.043[0.75-1.45]	0.565[0.27-1.17]
UA(per 1 μmol/L)	1.007[1.0-1.02]	1.007[1.0-1.01]	1.006[1.0-1.01]
Phosphorus(per 1 mmol/L)	0.051[0.01-0.38]	0.049[0.01-0.28]	0.016[0.0-0.16]
Calcium(per 1 mmol/L)	0.144[0.03-0.71]	0.246[0.04-1.36]	0.011[0.0-0.17]

HR, hazard ratio; 95% CI, 95% confidence interval; HD, hemodialysis; PD, peritoneal dialysis; BMI, body mass index; CAD, coronary artery disease; CCI, Charlson Comorbidity Index (calculated without age component); EF, left ventricular ejection fraction; FS, left ventricular fractional shortening;HDLC, high-density lipoprotein cholesterol; LDLC, low-density lipoprotein cholesterol;UA, uric acid; Model 1: Univariate analysis; Model 2: Multivariate analysis adjusted for Age at dialysis initiation; Model 3: Multivariate analysis adjusted for Age at dialysis initiation, Calcium and Phosphorus.

Table S9: Univariate to multivariate competing risk Cox regression model analysis of risk factors for cardiovascular disease death in a population with comorbid heart failure disease.

	**Model1 HR [95%CI]**	**Model2 HR [95%CI]**	**Model3 HR [95%CI]**	**Model4 HR [95%CI]**
Dialysis type(PD vs. HD)	0.927[0.43-1.99]	0.918[0.43-1.94]	1.015[0.48-2.16]	0.943[0.43-2.08]
Sex(female vs. male)	0.651[0.31-1.36]	0.671[0.32-1.39]	0.617[0.29-1.31]	0.481[0.23-1.02]
Age at dialysis initiation(per 1 year)	1.034[0.98-1.09]	1.037[0.98-1.1]	1.023[0.97-1.08]	1.019[0.96-1.08]
BMI(per 1kg/m2)	1.038[1.01-1.07]	1.038[1.01-1.07]	1.035[1.01-1.06]	1.034[1.0-1.06]
Diabetes mellitus(Yes vs. No)	2.233[0.89-5.63]	2.081[0.82-5.28]	1.913[0.74-4.93]	1.759[0.69-4.48]
CAD heart(Yes vs. No)	2.366[1.08-5.2]	2.357[1.07-5.18]	2.357[1.07-5.18]	2.512[1.14-5.54]
CCI without age(per 1 point)	1.184[0.91-1.55]	1.144[0.87-1.5]	1.106[0.84-1.45]	1.146[0.88-1.49]
EF(per 1%)	0.974[0.94-1.0]	0.976[0.95-1.01]	0.985[0.95-1.02]	0.986[0.96-1.02]
FS(per 1%)	0.959[0.91-1.01]	0.962[0.91-1.01]	0.974[0.92-1.03]	0.962[0.92-1.01]
Hemoglobin(per 1g/L)	1.021[1.01-1.04]	1.021[1.01-1.03]	1.022[1.01-1.04]	1.022[1.01-1.04]
Plasma albumin(per 1g/L)	1.052[0.99-1.12]	1.052[0.99-1.12]	1.048[0.98-1.12]	1.008[0.93-1.09]
Total cholesterol(per 1 mmol/L)	1.142[0.82-1.59]	1.166[0.84-1.63]	1.173[0.87-1.58]	1.262[0.9-1.77]
Triglyceride(per 1 mmol/L)	0.840[0.58-1.22]	0.866[0.62-1.22]	0.816[0.52-1.29]	0.774[0.44-1.35]
HDLC(per 1 mmol/L)	2.091[0.5-8.72]	2.093[0.48-9.15]	2.629[0.51-13.5]	2.056[0.42-9.95]
LDLC(per 1 mmol/L)	1.259[0.92-1.72]	1.289[0.94-1.76]	1.279[0.96-1.71]	1.422[1.04-1.94]
UA(per 1 μmol/L)	0.998[1.0-1.0]	0.999[1.0-1.0]	0.999[1.0-1.0]	1.001[1.0-1.0]
Phosphorus(per 1 mmol/L)	1.297[0.65-2.61]	1.289[0.63-2.62]	1.514[0.71-3.25]	1.441[0.62-3.37]
Calcium(per 1 mmol/L)	1.467[0.51-4.26]	1.376[0.45-4.24]	1.155[0.38-3.54]	1.100[0.34-3.52]

HR, hazard ratio; 95% CI, 95% confidence interval; HD, hemodialysis; PD, peritoneal dialysis; BMI, body mass index; CAD, coronary artery disease; CCI, Charlson Comorbidity Index (calculated without age component); EF, left ventricular ejection fraction; FS, left ventricular fractional shortening;HDLC, high-density lipoprotein cholesterol; LDLC, low-density lipoprotein cholesterol;UA, uric acid; Model 1: Univariate analysis; Model 2: Multivariate analysis adjusted for BMI; Model 3:Multivariate analysis adjusted for BMI, CAD; Model 4: Multivariate analysis adjusted for BMI, CAD and Hemoglobin.

Table S10: Univariate to multivariate Cox regression model analysis of risk factors for all-cause mortality in a population with

combined cerebrovascular disease.

	Model1 HR [95%CI]	Model2 HR [95%CI]	Model3 HR [95%CI]	Model4 HR [95%CI]	Model5 HR [95%CI]
Dialysis type(PD vs. HD)	0.764[0.47-1.25]	0.690[0.42-1.14]	0.639[0.38-1.07]	0.637[0.38-1.07]	0.527[0.31-0.91]
Sex(female vs. male)	1.029[0.63-1.67]	1.289[0.77-2.14]	1.299[0.78-2.16]	1.281[0.76-2.15]	1.138[0.67-1.94]
Age at dialysis initiation(per 1 year)	1.017[0.98-1.05]	1.032[0.99-1.07]	1.033[1.0-1.07]	1.040[1.0-1.08]	1.040[1.0-1.08]
BMI(per 1kg/m2)	1.009[0.92-1.11]	0.977[0.88-1.08]	0.956[0.86-1.06]	0.953[0.86-1.06]	0.974[0.87-1.09]
HBP(per 1mmHg)	0.798[0.19-3.28]	0.657[0.16-2.74]	0.683[0.16-2.85]	0.695[0.17-2.92]	0.512[0.12-2.22]
CAD(Yes vs. No)	1.602[0.97-2.65]	1.487[0.9-2.47]	1.469[0.88-2.44]	1.583[0.92-2.72]	1.515[0.88-2.6]
CCI without age(per 1 point)	1.150[0.99-1.34]	1.088[0.92-1.28]	1.088[0.92-1.28]	1.065[0.89-1.28]	1.054[0.87-1.27]
EF(per 1%)	0.985[0.96-1.01]	0.987[0.96-1.01]	0.986[0.96-1.01]	0.983[0.96-1.01]	0.983[0.96-1.01]
FS(per 1%)	0.970[0.93-1.01]	0.972[0.94-1.01]	0.971[0.94-1.01]	0.967[0.93-1.01]	0.968[0.93-1.01]
Hemoglobin(per 1g/L)	1.000[0.99-1.01]	1.001[0.99-1.01]	1.002[0.99-1.01]	1.002[0.99-1.02]	1.003[0.99-1.02]
Plasma albumin(per 1g/L)	0.970[0.92-1.02]	0.986[0.93-1.04]	0.992[0.94-1.05]	0.991[0.94-1.05]	0.970[0.91-1.03]
Total cholesterol(per 1 mmol/L)	1.109[0.9-1.36]	1.175[0.97-1.42]	1.154[0.94-1.41]	1.246[0.93-1.67]	1.303[0.97-1.75]
Triglyceride(per 1 mmol/L)	1.324[0.99-1.77]	1.141[0.83-1.57]	1.083[0.77-1.53]	1.087[0.77-1.53]	1.127[0.8-1.6]
HDLC(per 1 mmol/L)	0.345[0.15-0.8]	0.345[0.15-0.8]	0.393[0.16-0.96]	0.429[0.17-1.11]	0.401[0.15-1.04]
LDLC(per 1 mmol/L)	1.179[0.91-1.53]	1.244[0.98-1.58]	1.219[0.95-1.56]	1.257[0.94-1.68]	1.313[0.97-1.77]
UA(per 1 μmol/L)	1.000[1.0-1.0]	1.000[1.0-1.0]	1.000[1.0-1.0]	1.000[1.0-1.0]	1.001[1.0-1.0]
Phosphorus(per 1 mmol/L)	0.843[0.47-1.51]	0.869[0.49-1.55]	0.928[0.51-1.67]	0.909[0.5-1.64]	0.940[0.52-1.69]
Calcium(per 1 mmol/L)	0.806[0.26-2.52]	0.905[0.31-2.68]	0.949[0.31-2.9]	1.007[0.31-3.31]	0.879[0.27-2.87]

HR, hazard ratio; 95%CI, 95% confidence interval; HD, hemodialysis; PD, peritoneal dialysis; BMI, body mass index; HBP, hypertension; CAD, coronary artery disease; CCI, Charlson Comorbidity Index (calculated without age component); EF, left ventricular ejection fraction; FS, left ventricular fractional shortening; HDLC, high-density lipoprotein cholesterol; LDLC, low-density lipoprotein cholesterol; UA, uric acid; Model 1: Univariate analysis; Model 2: Multivariate analysis adjusted for HDLC; Model 3: Multivariate analysis adjusted for HDLC and CCI without age; Model 4: Multivariate analysis adjusted for HDLC , CCI without age,Total cholesterol and Plasma albumin; Model 5: Multivariate analysis adjusted for HDLC, CCI without age,Total cholesterol, Plasma albumin and age at dialysis initiation.

Table S11: Univariate to multivariate competing risk Cox regression model analysis of risk factors for hemorrhagic death in a population with comorbid cerebrovascular disease.

	Model1 HR [95%CI]	Model2 HR [95%CI]	Model3 HR [95%CI]
Dialysis type(PD vs. HD)	57717.015[20751.44-160531.2]	111195.446[18265.69-676920.72]	184875.937[13520.1-2528022.24]
Sex(female vs. male)	0.463[0.05-4.21]	0.247[0.07-0.87]	0.247[0.07-0.87]
Age at dialysis initiation(per 1 year)	0.952[0.87-1.04]	0.812[0.7-0.95]	0.837[0.73-0.96]
BMI(per 1kg/m2)	0.650[0.45-0.94]	0.671[0.47-0.97]	0.599[0.4-0.89]
Diabetes mellitus(Yes vs. No)	0.476[0.07-3.33]	1.288[0.15-11.43]	1.498[0.13-16.64]
CAD heart(Yes vs. No)	1.943[0.29-12.98]	0.628[0.04-10.69]	0.953[0.06-14.23]
CCI without age(per 1 point)	0.890[0.72-1.1]	0.860[0.52-1.44]	0.858[0.51-1.45]
EF(per 1%)	0.935[0.88-1.0]	0.973[0.9-1.05]	0.984[0.93-1.04]
FS(per 1%)	0.905[0.82-1.0]	0.856[0.59-1.25]	0.806[0.6-1.09]
Hemoglobin(per 1g/L)	0.937[0.88-1.0]	0.951[0.89-1.02]	0.946[0.9-0.99]
Plasma albumin(per 1g/L)	1.100[0.95-1.27]	1.192[0.99-1.43]	1.216[0.96-1.54]
Total cholesterol(per 1 mmol/L)	1.078[0.75-1.55]	1.147[0.61-2.17]	1.415[0.67-2.98]
Triglyceride(per 1 mmol/L)	1.099[0.61-1.96]	0.927[0.39-2.22]	1.823[0.49-6.77]

HDLC(per 1 mmol/L)	0.050[0.0-1.13]	0.018[0.0-1.09]	0.017[0.0-0.48]
LDLC(per 1 mmol/L)	1.075[0.75-1.55]	1.348[0.64-2.82]	1.542[0.7-3.39]
UA(per 1 μmol/L)	1.014[1.01-1.02]	1.027[1.01-1.04]	1.032[1.02-1.05]
Phosphorus(per 1 mmol/L)	0.011[0.0-7.66]	0.105[0.0-87.17]	0.019[0.0-1372.18]
Calcium(per 1 mmol/L)	3.678[0.09-144.04]	1.783[0.11-29.54]	4.526[0.21-97.92]

HR, hazard ratio; 95% CI, 95% confidence interval; HD, hemodialysis; PD, peritoneal dialysis; BMI, body mass index; CAD, coronary artery disease; CCI, Charlson Comorbidity Index (calculated without age component); EF, left ventricular ejection fraction; FS, left ventricular fractional shortening;HDLC, high-density lipoprotein cholesterol; LDLC, low-density lipoprotein cholesterol; UA, uric acid; Model 1: Univariate analysis; Model 2: Multivariate analysis adjusted for sex and dialysis initiation age; Model 3: Multivariate analysis adjusted for sex, dialysis initiation age and CCI without age.

Table S12: Univariate to multivariate competing risk Cox regression model analysis of risk factors for cerebrovascular disease death in a population with comorbid cerebrovascular disease.

	**Model1 HR [95%CI]**	**Model2 HR [95%CI]**	**Model3 HR [95%CI]**	**Model4 HR [95%CI]**
Dialysis type(PD vs. HD)	2.271[0.24-21.08]	3.448[0.3-39.76]	1.006[0.11-9.52]	9.320[0.1-830.25]
Sex(female vs. male)	0.457[0.05-4.19]	0.320[0.04-2.74]	0.727[0.07-7.73]	1.236[0.09-16.18]
Age at dialysis initiation(per 1 year)	0.946[0.9-1.0]	0.943[0.89-1.0]	0.969[0.91-1.03]	0.921[0.85-1.0]
BMI(per 1kg/m2)	0.911[0.82-1.02]	0.924[0.8-1.07]	0.868[0.71-1.07]	0.945[0.77-1.16]
Diabetes mellitus(Yes vs. No)	0.156[0.02-1.54]	0.170[0.0-9.95]	0.005[0.0-2.1]	0.001[0.0-0.02]
CAD heart(Yes vs. No)	0.642[0.07-5.77]	0.955[0.09-9.88]	1.013[0.04-27.22]	0.895[0.03-30.8]
CCI without age(per 1 point)	0.676[0.5-0.92]	0.652[0.47-0.9]	0.478[0.28-0.82]	0.304[0.16-0.56]
EF(per 1%)	0.971[0.94-1.01]	0.962[0.92-1.01]	0.990[0.89-1.1]	0.985[0.85-1.13]
FS(per 1%)	0.949[0.9-1.0]	0.933[0.87-1.0]	0.956[0.81-1.13]	0.944[0.76-1.17]
Hemoglobin(per 1g/L)	0.953[0.87-1.05]	0.939[0.87-1.02]	0.920[0.86-0.99]	0.920[0.86-0.99]
Plasma albumin(per 1g/L)	1.076[0.92-1.26]	1.064[0.92-1.23]	1.087[0.88-1.34]	1.229[0.99-1.53]
Total cholesterol(per 1 mmol/L)	1.142[0.7-1.86]	1.179[0.66-2.12]	1.164[0.54-2.52]	1.620[0.6-4.34]
Triglyceride(per 1 mmol/L)	0.961[0.43-2.15]	1.244[0.35-4.36]	1.829[0.43-7.73]	2.122[0.34-13.15]
HDLC(per 1 mmol/L)	0.244[0.0-20.25]	0.199[0.01-3.51]	0.244[0.02-3.81]	0.253[0.0-14.08]
LDLC(per 1 mmol/L)	1.023[0.58-1.8]	1.046[0.53-2.06]	0.966[0.36-2.59]	1.489[0.48-4.6]
UA(per 1 μmol/L)	1.010[1.0-1.02]	1.010[1.0-1.02]	1.011[1.0-1.02]	1.011[1.01-1.02]
Phosphorus(per 1 mmol/L)	1.612[0.13-19.57]	1.514[0.09-24.98]	0.828[0.11-6.52]	1.140[0.24-5.37]
Calcium(per 1 mmol/L)	0.251[0.0-17.02]	0.251[0.01-7.64]	0.367[0.05-2.83]	0.375[0.06-2.42]

HR, hazard ratio; 95% CI, 95% confidence interval; HD, hemodialysis; PD, peritoneal dialysis; BMI, body mass index; CAD, coronary artery disease; CCI, Charlson Comorbidity Index (calculated without age component); EF, left ventricular ejection fraction; FS, left ventricular fractional shortening;HDLC, high-density lipoprotein cholesterol; LDLC, low-density lipoprotein cholesterol;UA, uric acid; Model 1: Univariate analysis; Model 2: Multivariate analysis adjusted for Age at dialysis initiation,CCI without age; Model 3: Multivariate analysis adjusted for Age at dialysis initiation,CCI without age, Plasma albumin and UA; Model 4: Multivariate analysis adjusted for Age at dialysis initiation, CCI without age, Plasma albumin,UA and Hemoglobin.

Table S13: Univariate to multivariate competing risk Cox regression model analysis of risk factors for cardiovascular disease death in a population with comorbid cerebrovascular disease.

	**Model1 HR [95%CI]**	**Model2 HR [95%CI]**	**Model3 HR [95%CI]**	**Model4 HR [95%CI]**
Dialysis type(PD vs. HD)	1.295[0.55-3.07]	1.060[0.47-2.41]	1.048[0.45-2.45]	0.976[0.44-2.15]

Sex(female vs. male)	0.500[0.2-1.28]	0.598[0.22-1.66]	0.586[0.21-1.64]	0.647[0.22-1.9]
Age at dialysis initiation(per 1 year)	1.008[0.95-1.07]	1.022[0.96-1.08]	1.007[0.95-1.07]	1.023[0.97-1.08]
BMI(per 1kg/m2)	1.353[1.15-1.6]	1.339[1.13-1.59]	1.286[1.06-1.56]	1.305[1.09-1.56]
Diabetes mellitus(Yes vs. No)	5.482[1.28-23.49]	3.943[0.76-20.51]	5.296[0.96-29.37]	4.719[0.87-25.53]
CAD heart(Yes vs. No)	1.852[0.81-4.25]	1.821[0.82-4.02]	2.016[0.81-4.99]	1.745[0.67-4.54]
CCI without age(per 1 point)	1.315[1.04-1.66]	1.170[0.94-1.45]	1.213[0.99-1.49]	1.252[0.99-1.58]
EF(per 1%)	0.968[0.93-1.01]	0.955[0.92-0.99]	0.950[0.91-0.99]	0.950[0.91-0.99]
FS(per 1%)	0.952[0.9-1.01]	0.935[0.88-0.99]	0.928[0.87-0.99]	0.937[0.72-1.22]
Hemoglobin(per 1g/L)	1.022[1.01-1.04]	1.018[1.0-1.04]	1.016[1.0-1.03]	1.017[1.0-1.03]
Plasma albumin(per 1g/L)	1.058[0.98-1.14]	1.055[0.98-1.14]	1.035[0.96-1.11]	1.022[0.94-1.11]
Total cholesterol(per 1 mmol/L)	0.944[0.59-1.5]	1.011[0.7-1.46]	1.005[0.66-1.52]	0.947[0.66-1.36]
Triglyceride(per 1 mmol/L)	1.110[0.63-1.95]	0.922[0.55-1.53]	0.869[0.49-1.53]	0.746[0.41-1.35]
HDLC(per 1 mmol/L)	0.591[0.16-2.2]	0.900[0.11-7.07]	0.885[0.1-7.59]	1.104[0.11-11.55]
LDLC(per 1 mmol/L)	0.995[0.56-1.77]	1.116[0.71-1.75]	1.162[0.68-1.98]	1.046[0.67-1.63]
UA(per 1 μmol/L)	1.002[1.0-1.0]	1.001[1.0-1.0]	1.002[1.0-1.0]	1.002[1.0-1.0]
Phosphorus(per 1 mmol/L)	1.261[0.56-2.85]	1.046[0.37-2.92]	1.226[0.42-3.6]	1.196[0.39-3.7]
Calcium(per 1 mmol/L)	1.174[0.24-5.65]	1.526[0.29-8.05]	1.054[0.14-7.99]	1.140[0.15-8.62]

HR, hazard ratio; 95% CI, 95% confidence interval; HD, hemodialysis; PD, peritoneal dialysis; BMI, body mass index; CAD, coronary artery disease; CCI, Charlson Comorbidity Index (calculated without age component); EF, left ventricular ejection fraction; FS, left ventricular fractional shortening;HDLC, high-density lipoprotein cholesterol; LDLC, low-density lipoprotein cholesterol;UA, uric acid; Model 1: Univariate analysis; Model 2: Multivariate analysis adjusted for BMI, CCI without age; Model 3: Multivariate analysis adjusted for BMI, CCI without age, Hemoglobin and Plasma albumin; Model 4: Multivariate analysis adjusted for BMI, CCI without age, Hemoglobin, Plasma albumin and EF.

Table S14: Univariate to multivariate Cox regression models analyzing risk factors for all-cause mortality in the comorbid diabetic population.

	**Model1 HR [95%CI]**	**Model2 HR [95%CI]**	**Model3 HR [95%CI]**
Dialysis type(PD vs. HD)	0.683[0.48-0.97]	0.689[0.48-0.98]	0.687[0.48-0.98]
Sex(female vs. male)	0.915[0.65-1.29]	0.920[0.65-1.3]	0.813[0.57-1.16]
Age at dialysis initiation(per 1 year)	1.023[1.0-1.05]	1.021[1.0-1.05]	1.016[0.99-1.04]
BMI(per 1kg/m2)	0.955[0.91-1.0]	0.955[0.91-1.0]	0.947[0.9-0.99]
HBP(per 1mmHg)	0.626[0.2-1.97]	0.628[0.2-1.98]	0.569[0.18-1.8]
CAD(Yes vs. No)	1.517[1.07-2.16]	1.636[1.15-2.34]	1.636[1.15-2.34]
CCI without age(per 1 point)	0.945[0.81-1.1]	0.970[0.83-1.13]	0.977[0.84-1.14]
EF(per 1%)	0.984[0.97-1.0]	0.982[0.96-1.0]	0.988[0.97-1.01]
FS(per 1%)	0.975[0.95-1.0]	0.972[0.95-1.0]	0.979[0.95-1.01]
Hemoglobin(per 1g/L)	0.999[0.99-1.01]	1.000[0.99-1.01]	1.001[0.99-1.01]
Plasma albumin(per 1g/L)	0.987[0.95-1.02]	0.991[0.96-1.03]	0.988[0.95-1.03]
Total cholesterol(per 1 mmol/L)	1.025[0.9-1.17]	1.020[0.89-1.17]	1.031[0.9-1.18]
Triglyceride(per 1 mmol/L)	1.062[0.96-1.18]	1.056[0.95-1.17]	1.055[0.95-1.17]
HDLC(per 1 mmol/L)	0.652[0.36-1.17]	0.680[0.38-1.22]	0.712[0.39-1.29]
LDLC(per 1 mmol/L)	1.058[0.9-1.25]	1.041[0.88-1.23]	1.049[0.89-1.24]
UA(per 1 μmol/L)	1.000[1.0-1.0]	1.000[1.0-1.0]	1.000[1.0-1.0]
Phosphorus(per 1 mmol/L)	0.810[0.57-1.15]	0.835[0.59-1.18]	0.866[0.61-1.23]
Calcium(per 1 mmol/L)	1.464[0.69-3.1]	1.689[0.81-3.54]	1.533[0.72-3.24]

HR, hazard ratio; 95%CI, 95% confidence interval; HD, hemodialysis; PD, peritoneal dialysis; BMI, body mass index; HBP, hypertension; CAD, coronary artery disease; CCI, Charlson Comorbidity Index (calculated without age component); EF, left ventricular ejection fraction; FS, left ventricular fractional shortening; HDLC, high-density lipoprotein cholesterol; LDLC, low-density lipoprotein cholesterol; UA, uric acid; Model 1: Univariate analysis;Model 2: Multivariate analysis adjusted for BMI; Model 3: Multivariate analysis adjusted for BMI and CAD.

Table S15: Univariate to multivariate competing risk Cox regression models analyzing risk factors for hemorrhagic death in the comorbid diabetes population.

	**Model1 HR [95%CI]**	**Model2 HR [95%CI]**	**Model3 HR [95%CI]**
Dialysis type(PD vs. HD)	3.964[0.48-32.53]	4.229[0.52-34.29]	4.286[0.53-34.73]
Sex(female vs. male)	0.464[0.09-2.34]	0.552[0.11-2.73]	0.676[0.12-3.75]
Age at dialysis initiation(per 1 year)	0.920[0.81-1.04]	0.930[0.82-1.05]	0.921[0.81-1.05]
BMI(per 1kg/m2)	0.851[0.68-1.07]	0.835[0.64-1.08]	0.838[0.65-1.08]
CAD heart(Yes vs. No)	0.764[0.15-3.84]	0.787[0.16-3.88]	0.797[0.16-3.92]
CCI without age(per 1 point)	0.724[0.38-1.38]	0.709[0.35-1.42]	0.704[0.35-1.41]
EF(per 1%)	0.993[0.93-1.06]	0.997[0.94-1.06]	0.996[0.94-1.05]
FS(per 1%)	1.042[0.99-1.1]	1.040[0.99-1.1]	1.039[0.99-1.1]
Hemoglobin(per 1g/L)	0.957[0.91-1.0]	0.958[0.92-1.0]	0.959[0.92-1.0]
Plasma albumin(per 1g/L)	0.986[0.89-1.09]	0.979[0.89-1.08]	0.977[0.88-1.08]
Total cholesterol(per 1 mmol/L)	0.903[0.72-1.13]	1.026[0.8-1.32]	1.363[0.91-2.04]
Triglyceride(per 1 mmol/L)	0.859[0.72-1.02]	0.701[0.54-0.91]	0.701[0.54-0.91]
HDLC(per 1 mmol/L)	0.082[0.01-0.79]	0.082[0.01-0.79]	0.052[0.0-0.64]
LDLC(per 1 mmol/L)	0.970[0.78-1.21]	1.130[0.84-1.51]	1.231[0.89-1.71]
UA(per 1 μmol/L)	1.005[1.0-1.01]	1.005[1.0-1.01]	1.005[1.0-1.01]
Phosphorus(per 1 mmol/L)	1.571[0.27-9.01]	1.476[0.33-6.54]	1.468[0.38-5.65]
Calcium(per 1 mmol/L)	0.116[0.01-2.35]	0.178[0.01-2.66]	0.176[0.01-3.47]

HR, hazard ratio; 95% CI, 95% confidence interval; HD, hemodialysis; PD, peritoneal dialysis; BMI, body mass index; CAD, coronary artery disease; CCI, Charlson Comorbidity Index (calculated without age component); EF, left ventricular ejection fraction; FS, left ventricular fractional shortening;HDLC, high-density lipoprotein cholesterol; LDLC, low-density lipoprotein cholesterol;UA, uric acid; Model 1: Univariate analysis; Model 2: Multivariate analysis adjusted for HDLC; Model 3: Multivariate analysis adjusted for HDLC and Triglyceride.

Table S16: Univariate to multivariate competing risk Cox regression models analyzing risk factors for cerebrovascular death in the comorbid diabetes population.

	**Model1 HR [95%CI]**	**Model2 HR [95%CI]**	**Model3 HR [95%CI]**	**Model4 HR [95%CI]**	**Model5 HR [95%CI]**
Dialysis type(PD vs. HD)	3.311[0.39-28.16]	3.536[0.42-29.61]	3.502[0.39-31.48]	9.783[3.41-28.11]	16.701[2.76-101.04]
Sex(female vs. male)	0.233[0.03-1.96]	0.286[0.03-2.43]	0.343[0.04-3.2]	0.093[0.02-0.35]	0.117[0.04-0.38]
Age at dialysis initiation(per 1 year)	0.869[0.75-1.01]	0.881[0.76-1.02]	0.898[0.78-1.04]	0.888[0.76-1.04]	0.912[0.79-1.06]
BMI(per 1kg/m2)	0.946[0.85-1.05]	0.939[0.83-1.06]	0.956[0.81-1.13]	0.985[0.79-1.23]	1.050[0.84-1.32]
CAD heart(Yes vs. No)	0.381[0.05-3.17]	0.402[0.05-3.36]	0.520[0.06-4.52]	0.182[0.05-0.71]	0.340[0.09-1.34]
CCI without age(per 1 point)	0.711[0.34-1.51]	0.696[0.3-1.6]	0.816[0.3-2.19]	0.715[0.3-1.71]	0.762[0.33-1.77]
EF(per 1%)	0.992[0.92-1.07]	0.996[0.93-1.06]	1.029[0.94-1.12]	0.900[0.84-0.96]	0.802[0.72-0.89]
FS(per 1%)	1.049[0.99-1.11]	1.047[0.99-1.11]	1.106[1.03-1.19]	1.287[1.12-1.48]	1.472[1.23-1.77]
Hemoglobin(per 1g/L)	0.964[0.92-1.01]	0.965[0.92-1.01]	0.952[0.89-1.02]	0.945[0.88-1.02]	0.942[0.89-1.0]

Plasma albumin(per 1g/L)	0.965[0.87-1.07]	0.959[0.87-1.06]	1.020[0.92-1.13]	1.029[0.93-1.14]	1.069[0.93-1.23]
Total cholesterol(per 1 mmol/L)	0.893[0.69-1.16]	1.040[0.78-1.39]	0.977[0.66-1.44]	0.910[0.53-1.57]	0.925[0.46-1.87]
Triglyceride(per 1 mmol/L)	0.873[0.73-1.04]	0.687[0.51-0.92]	0.652[0.39-1.08]	0.691[0.44-1.09]	0.689[0.33-1.45]
HDLC(per 1 mmol/L)	0.042[0.0-0.58]	0.042[0.0-0.58]	0.077[0.01-0.89]	0.096[0.0-2.1]	0.264[0.01-5.74]
LDLC(per 1 mmol/L)	0.971[0.76-1.24]	1.171[0.84-1.63]	1.094[0.73-1.64]	0.966[0.51-1.82]	0.853[0.38-1.92]
UA(per 1 μmol/L)	1.005[1.0-1.01]	1.005[1.0-1.01]	1.004[1.0-1.01]	1.004[0.99-1.01]	1.004[0.99-1.01]
Phosphorus(per 1 mmol/L)	2.365[0.52-10.75]	2.048[0.56-7.45]	1.321[0.27-6.46]	1.324[0.27-6.39]	1.684[0.26-10.92]
Calcium(per 1 mmol/L)	0.031[0.01-0.18]	0.056[0.01-0.28]	0.056[0.01-0.28]	0.010[0.0-0.12]	0.021[0.0-0.49]

HR, hazard ratio; 95% CI, 95% confidence interval; HD, hemodialysis; PD, peritoneal dialysis; BMI, body mass index; CAD, coronary artery disease; CCI, Charlson Comorbidity Index (calculated without age component); EF, left ventricular ejection fraction; FS, left ventricular fractional shortening;HDLC, high-density lipoprotein cholesterol; LDLC, low-density lipoprotein cholesterol;UA, uric acid; Model 1: Univariate analysis; Model 2: Multivariate analysis adjusted for HDLC; Model 3: Multivariate analysis adjusted for HDLC and Calcium; Model 4: Multivariate analysis adjusted for HDLC, Calcium, EF and FS; Model 5: Multivariate analysis adjusted for HDLC, Calcium, EF, FS, CAD and sex.

Table S17: Univariate to multivariate competing risk Cox regression models analyzing risk factors for cardiovascular disease death in the comorbid diabetes population.

	**Model1 HR [95%CI]**	**Model2 HR [95%CI]**	**Model3 HR [95%CI]**	**Model4 HR [95%CI]**	**Model5 HR [95%CI]**
Dialysis type(PD vs. HD)	0.893[0.52-1.55]	0.891[0.52-1.54]	0.935[0.54-1.62]	0.959[0.55-1.66]	1.01[0.58-1.77]
Sex(female vs. male)	0.803[0.46-1.39]	0.804[0.46-1.39]	0.738[0.42-1.31]	0.748[0.42-1.32]	0.769[0.43-1.37]
Age at dialysis initiation(per 1 year)	1.014[0.97-1.06]	1.017[0.98-1.06]	1.013[0.97-1.06]	1.01[0.97-1.05]	1.018[0.97-1.06]
BMI(per 1kg/m2)	1.044[1.01-1.08]	1.044[1.01-1.08]	1.034[1.0-1.06]	1.032[1.0-1.07]	1.028[1.0-1.06]
CAD heart(Yes vs. No)	2.034[1.19-3.48]	1.937[1.12-3.35]	1.937[1.12-3.35]	2.026[1.17-3.5]	1.849[1.04-3.28]
CCI without age(per 1 point)	1.002[0.79-1.27]	0.977[0.77-1.23]	0.966[0.76-1.22]	0.991[0.79-1.24]	1.000[0.79-1.26]
EF(per 1%)	0.971[0.95-1.0]	0.975[0.95-1.0]	0.983[0.96-1.01]	0.986[0.96-1.01]	0.986[0.96-1.01]
FS(per 1%)	0.952[0.92-0.99]	0.957[0.92-1.0]	0.967[0.93-1.01]	0.965[0.93-1.0]	0.927[0.85-1.01]
Hemoglobin(per 1g/L)	1.015[1.0-1.03]	1.015[1.0-1.03]	1.016[1.0-1.03]	1.016[1.0-1.03]	1.015[1.0-1.03]
Plasma albumin(per 1g/L)	1.032[0.97-1.1]	1.027[0.96-1.09]	1.023[0.96-1.09]	0.996[0.93-1.07]	0.994[0.93-1.06]
Total cholesterol(per 1 mmol/L)	1.091[0.87-1.37]	1.103[0.88-1.38]	1.095[0.89-1.35]	1.088[0.88-1.34]	1.118[0.89-1.4]
Triglyceride(per 1 mmol/L)	1.032[0.9-1.19]	1.037[0.9-1.19]	1.02[0.88-1.18]	1.007[0.86-1.17]	1.011[0.87-1.18]
HDLC(per 1 mmol/L)	0.978[0.4-2.37]	0.970[0.39-2.4]	1.054[0.43-2.6]	0.987[0.42-2.32]	1.048[0.44-2.48]
LDLC(per 1 mmol/L)	1.185[0.9-1.56]	1.220[0.93-1.61]	1.210[0.93-1.57]	1.229[0.95-1.6]	1.25[0.96-1.63]
UA(per 1 μmol/L)	1.001[1.0-1.0]	1.001[1.0-1.0]	1.001[1.0-1.0]	1.002[1.0-1.0]	1.002[1.0-1.0]
Phosphorus(per 1 mmol/L)	0.965[0.54-1.73]	0.949[0.52-1.72]	0.982[0.53-1.82]	0.973[0.5-1.88]	0.948[0.49-1.84]
Calcium(per 1 mmol/L)	1.401[0.52-3.75]	1.271[0.46-3.54]	1.045[0.38-2.85]	0.863[0.31-2.41]	0.964[0.35-2.62]

HR, hazard ratio; 95% CI, 95% confidence interval; HD, hemodialysis; PD, peritoneal dialysis; BMI, body mass index; CAD, coronary artery disease; CCI, Charlson Comorbidity Index (calculated without age component); EF, left ventricular ejection fraction; FS, left ventricular fractional shortening;HDLC, high-density lipoprotein cholesterol; LDLC, low-density lipoprotein cholesterol;UA, uric acid; Model 1: Univariate analysis; Model 2: Multivariate analysis adjusted for BMI; Model 3: Multivariate analysis adjusted for BMI, CAD; Model 4: Multivariate analysis adjusted for BMI,CAD and Hemoglobin; Model 5: Multivariate analysis adjusted for BMI, CAD, Hemoglobin, EF.

Figure S1: Cumulative incidence of all-cause mortality in the overall population and comparison with that in hemodialysis and peritoneal dialysis groups.

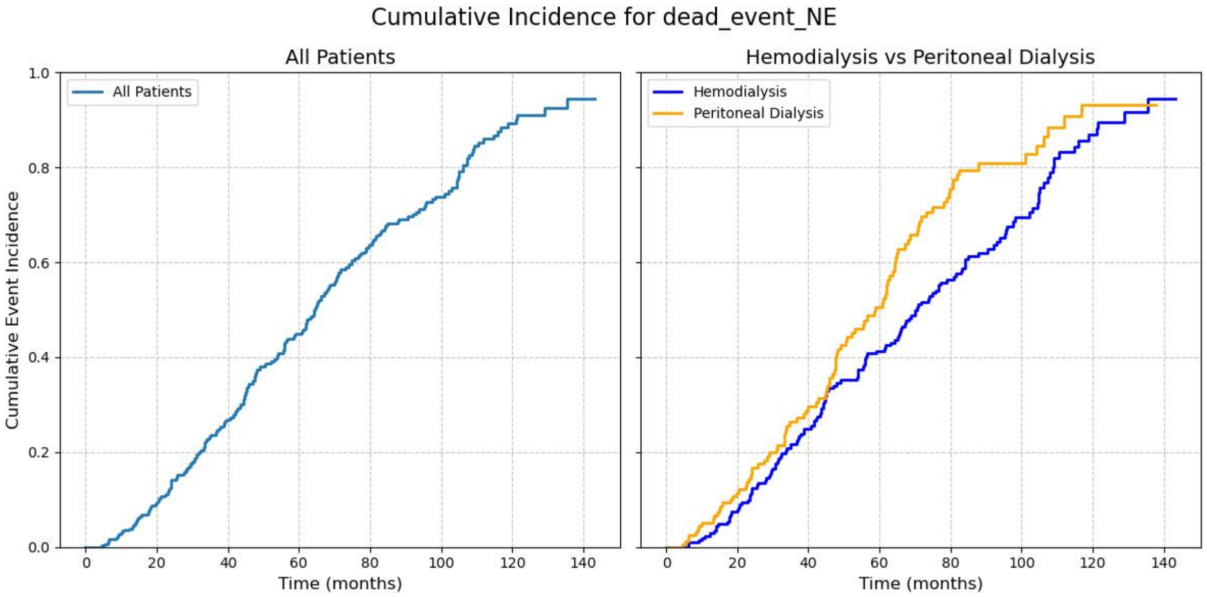

Figure S2: Cumulative incidence of death from infectious disease in the overall population and comparison with that in hemodialysis and peritoneal dialysis groups.

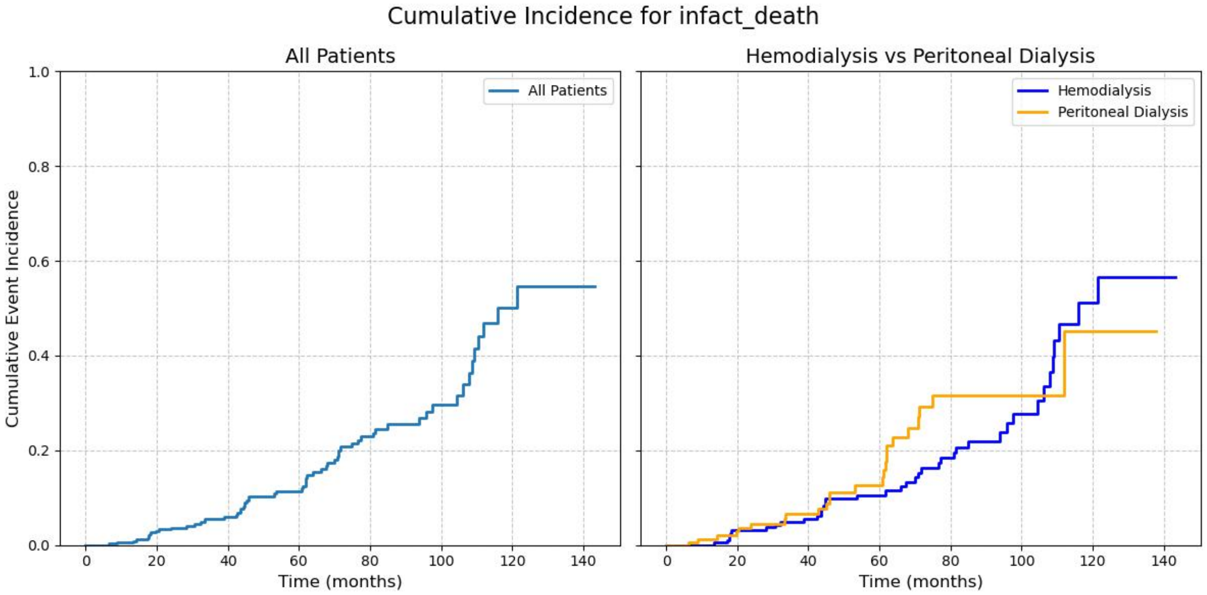

Figure S3: Cumulative incidence of death from cardiovascular disease in the overall population and comparison with that in hemodialysis and peritoneal dialysis groups.

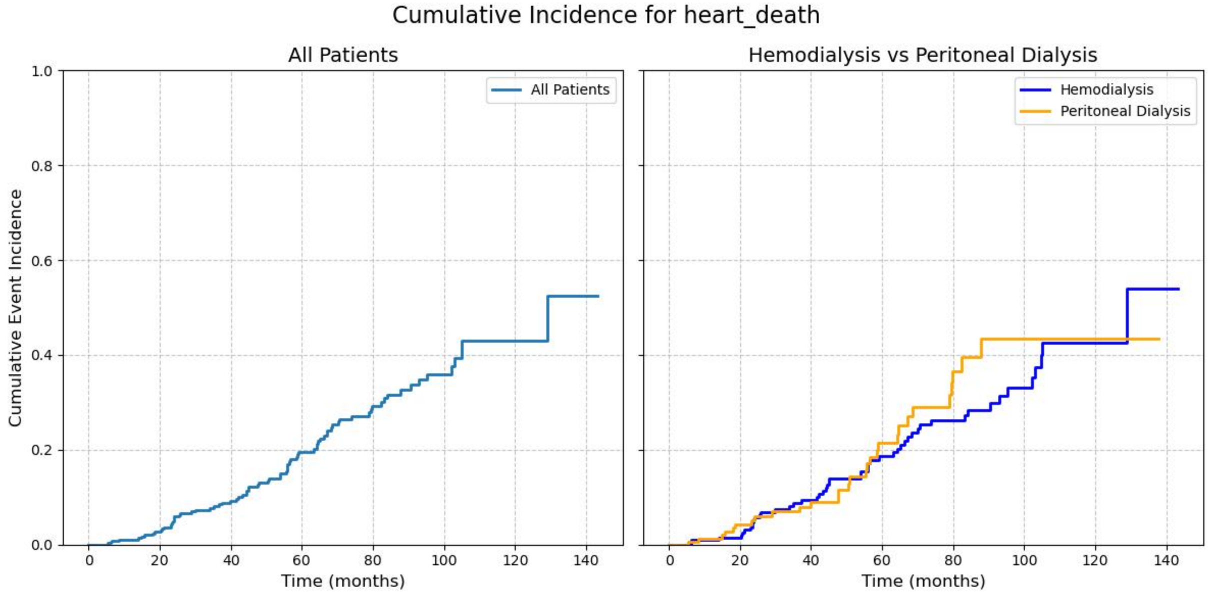

Figure S4: Cumulative incidence of death from cerebrovascular disease in the overall population and comparison with that in hemodialysis and peritoneal dialysis groups.

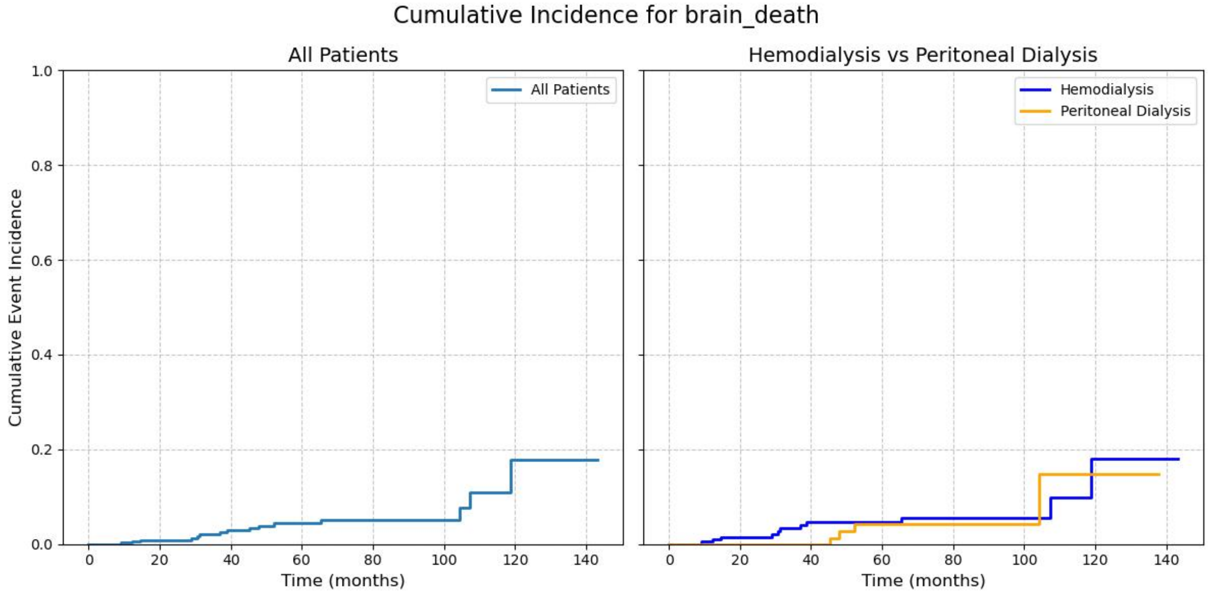

Figure S5: Cumulative incidence of death from hemorrhagic disease in the overall population and comparison with that in hemodialysis and peritoneal dialysis groups.

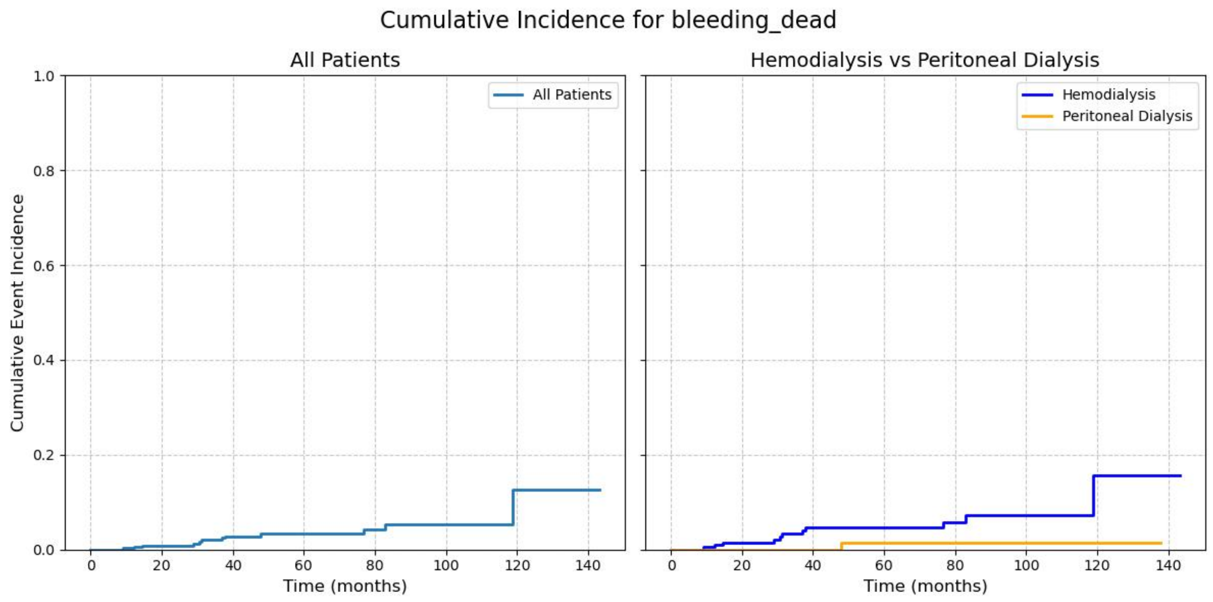

Figure S6: Cumulative incidence of death from other disease in the overall population and comparison with that in hemodialysis and peritoneal dialysis groups.

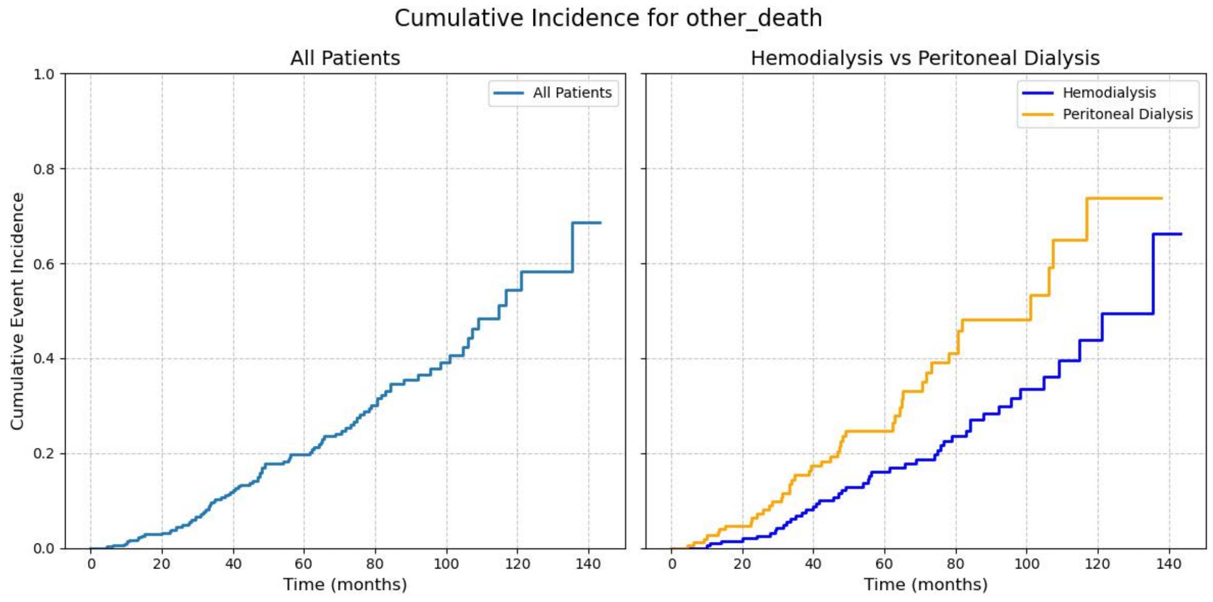

Figure S7: Survival Comparison Between Groups With and Without Heart Failure in the Overall Patients, Hemodialysis Patients, and Peritoneal Dialysis Patients.

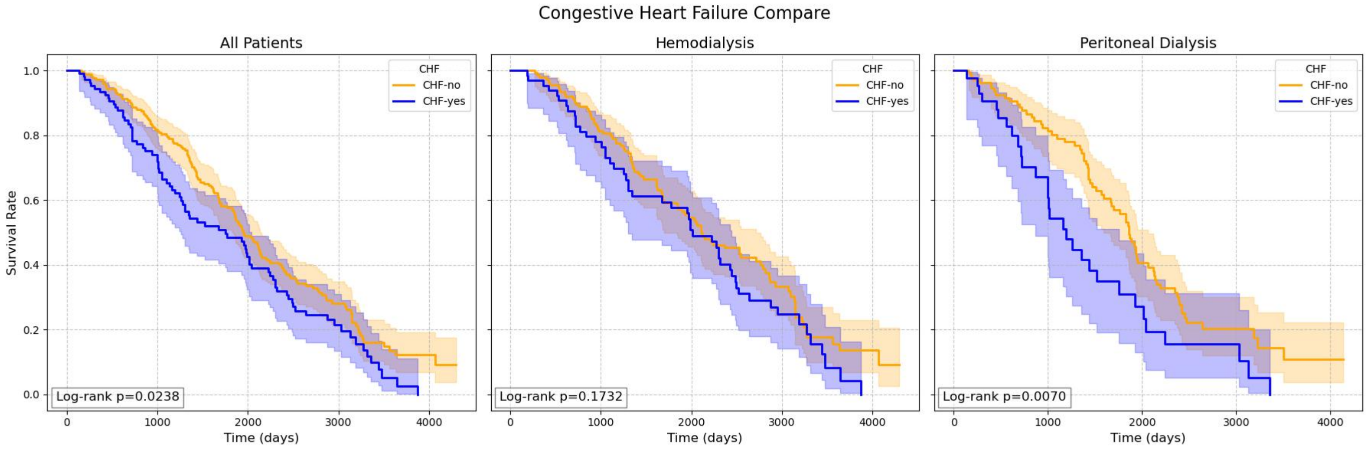

CHF-no,uncombined chronic heart failure group; CHF-yes, combining the chronic heart failure group.

Figure S8: Survival Comparison Between Groups With and Without cerebrovascular disease in the Overall Patients, Hemodialysis Patients, and Peritoneal Dialysis Patients.

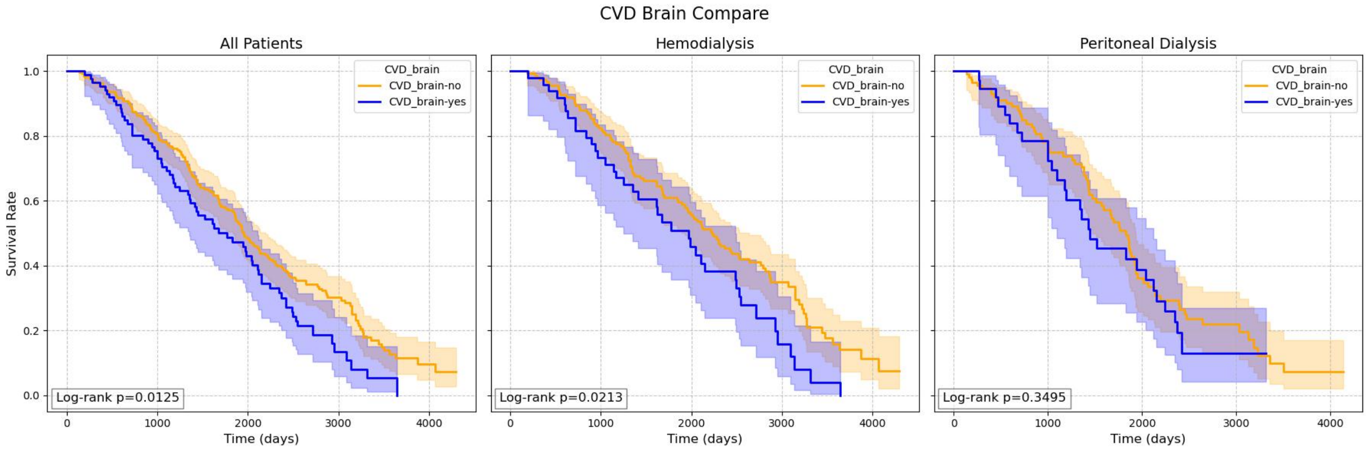

CHF-no,uncombined cerebrovascular disease group; CHF-yes, combining cerebrovascular disease group.

Figure S9: Survival Comparison Between Groups With and Without Diabetes mellitus in the Overall Patients, Hemodialysis Patients, and Peritoneal Dialysis Patients.

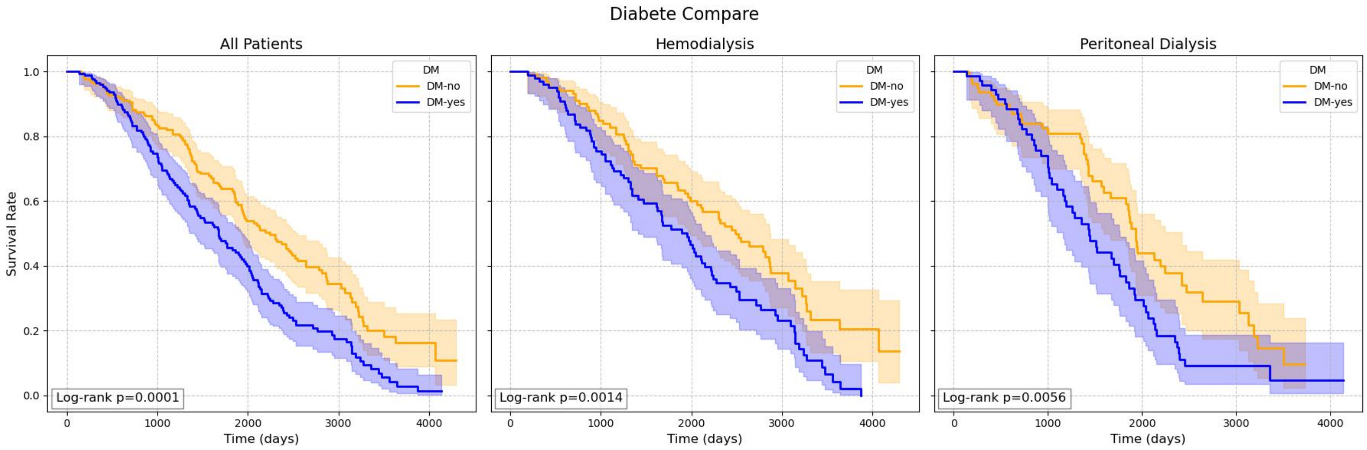

CHF-no,uncombined diabetes mellitus group; CHF-yes, combining diabetes mellitus group.
