# Supplementary material for: Hemodialysis versus peritoneal dialysis in elderly ESRD patients: a retrospective cohort study on survival and the role of comorbidities
Source: BMC Nephrol. 2026 Jan 30;27:142. doi: 10.1186/s12882-026-04783-7 (PMC12930720; doi:10.1186/s12882-026-04783-7)
Supplement: Supplementary file 2 — Supplementary Material 2 [file 12882_2026_4783_MOESM2_ESM.pdf]

SPRINGER NATURE

Change of authorship request form - Journals (pre-acceptance)

Important information. Please read.

- This form should be used by authors to request any change in authorship (adding/deleting authors) including changes in corresponding authors. This form should not be used for name changes. Please fully complete all sections. Use black ink and block capitals and provide each author's full name with the given name first followed by the family name.

- By signing this declaration, all authors guarantee that the order of the authors are in accordance with their scientific contribution, if applicable as different conventions apply per discipline, and that only authors have been added who made a meaningful contribution to the work.

- Please note, in author collaborations where there is formal agreement for representing the collaboration, it is sufficient for the representative or legal guarantor (usually the corresponding author) to complete and sign the Authorship Change Form on behalf of all authors, **next to the added/removed author(s)**. **(Complete Section 3, followed by Section 6.)**
  In author collaborations where there is no formal agreement for representing the collaboration and **there are more than 10 authors, one** may sign for all, provided the signer appends correspondence that attests that each of the authors have agreed to the change **and the added/removed authors sign the form**. **(Complete Section 3, followed by Section 6.)**

- Please note, we cannot investigate or mediate any authorship disputes. If you are unable to obtain agreement from all authors (including those who you wish to be removed) you must refer the matter to your institution(s) for investigation. Please inform us if you need to do this.

- If you are not able to return a fully completed form within **30 days** of the date that it was sent to the author requesting the change, we may have to withdraw your manuscript. We cannot publish manuscripts where authorship has not been agreed by all authors (including those who have been removed).

- Incomplete forms will be rejected.

- Please return/upload this form, fully completed, to the Journals Editorial Office. The Journal and/or Publisher will consider the information you have provided to decide whether to approve the proposed change in authorship. We may decide to contact your institution for more information or undertake a further investigation, if appropriate, before making a final decision.

SPRINGER NATURE

Change of authorship request form - Journals (pre-acceptance)

Section 1: Please provide the current title of manuscript

Manuscript ID no.: 883986d2-d0b6-44cd-8c1b-5a305C528de5

Title: Hemodialysis versus peritoneal dialysis in elderly ESRD patients: A retrospective cohort study on survival and the role of comorbidities

Section 2: Please provide the previous authorship, in the order shown on the manuscript before the changes were introduced. Please indicate the corresponding author by adding (CA) behind the name.

	First name(s)	Family name	ORCID or SCOPUS id, if available
1st author	Ruizhi	Yu	
2nd author	Yunfei	Yan	
3rd author	Junjie	Lin	
4th author	Youqing	Cai	
5th author	Lixin	Wang	
6th author	Xusheng	Liu	
7th author	Zhiren(CA)	He	

Please use an additional sheet if there are more than 10 authors.

SPRINGER NATURE Springer Nature is one of the world's leading global research, educational and professional publishers, created in May 2015 through the combination of Nature Publishing Group, Palgrave Macmillan, Macmillan Education and Springer Science+Business Media.

Change of authorship request form - Journals (pre-acceptance)

Section 3: Please provide a justification for change. Please use this section to explain your reasons for changing the authorship of your manuscript, e.g. what necessitated the change in authorship? Please refer to the (journal) policy pages for more information about authorship. Please explain why omitted authors were not originally included and/or why authors were removed on the submitted manuscript.

Hucai Li did not participate in the initial stages of the research or the drafting of the manuscript and therefore was not included among the original authors. As a new author has been added to the submission, an explanation is required: Hucai Li participated in revising the manuscript, with a specific focus on modifying the discussion section. He also assisted in completing the Variance Inflation Factor (VIF) analysis for variables in the multivariable Cox regression models, which has been included in the Supplementary materials. During this stage, the database was further refined based on his contributions, and all authors acknowledge that Dr Li made significant contributions during the revision of the paper. Accordingly, Dr. Li has been listed as the fourth author.

Section 4: Proposed new authorship. Please provide your new authorship list in the order you would like it to appear on the manuscript. Please indicate the corresponding author by adding (CA) behind the name. If the Corresponding Author has changed, please indicate the reason under section 3.

	First name(s)	Family name (this name will appear in full on the final publication and will be searchable in various abstract and indexing databases)	Affiliated institute	E-mail address
1st author	Ruizhi	Yu	The Second Clinical College of Guangzhou University of Chinese Medicine, guangzhou, China	20231120970@stu.gzucm.edu.cn
2nd author	Yunfei	Yan	The Second Clinical College of Guangzhou University of Chinese Medicine, guangzhou, China	15521143043@163.com
3rd author	Junjie	Lin	Guangdong Provincial Hospital of Traditional Chinese Medicine, Guangzhou, China, Department of Nephrology	linjunjie2010@163.com
4th author	Hucai	Li	Guangdong Provincial Hospital of Traditional Chinese Medicine, Guangzhou, China, Department of Nephrolog	lhc9034@163.com
5th author	Youqing	Cai	Guangdong Provincial Hospital of Traditional Chinese Medicine, Guangzhou, China, Department of Nephrolog	cyq1050@163.com
6th author	Lixin	Wang	Guangdong Provincial Hospital of Traditional Chinese Medicine, Guangzhou, China, Department of Nephrolog	wanglixin1210@163.com
7th author	Xusheng	Liu	Guangdong Provincial Hospital of Traditional Chinese Medicine, Guangzhou, China, Department of Nephrolog	liuxu801@126.com
8th author	Zhiren(CA)	He	Guangdong Provincial Hospital of Traditional Chinese Medicine, Guangzhou, China, Department of Nephrolog	he.zhiren@gzucm.edu.cn

Please use an additional sheet if there are more than 10 authors.

SPRINGER NATURE Springer Nature is one of the world's leading global research, educational and professional publishers, created in May 2015 through the combination of Nature Publishing Group, Palgrave Macmillan, Macmillan Education and Springer Science+Business Media.

Change of authorship request form - Journals (pre-acceptance)

Section 5: Author contribution, Acknowledgement and Disclosures. Please use this section to provide a new disclosure statement and, if appropriate, acknowledge any contributors who have been removed as authors and ensure you state what contribution any new authors made (if applicable per the journal or book (series) policy). Please ensure these are updated in your manuscript - after approval of the change(s) - as our production department will not transfer the information in this form to your manuscript.

New acknowledgements:

Not applicable

New Disclosures (financial and non-financial interests, funding):

Not applicable

New Author Contributions statement (if applicable per the journal policy):

Hucai Li participated in revising the manuscript, with a specific focus on modifying the discussion section.He also assisted in completing the Variance Inflation Factor (VIF) analysis for variables in the multivariable Cox regression models, which has been included in the Supplementary materials. During this stage, the database was further refined based on his contributions.

State 'Not applicable' if there are no new authors.

SPRINGER NATURE

Change of authorship request form - Journals (pre-acceptance)

Section 6: Declaration of agreement. ***All*** **authors, unchanged, new and removed** ***must*** **sign this declaration.**
(NB: Please print the form, (docu)-sign and return/upload a scanned copy. Please note that signatures that have been inserted as an image file are acceptable as long as it is handwritten. Typed names in the signature box are unacceptable.) * **Please delete as appropriate. Delete all of the bold if you were on the original authorship list and are remaining as an author.**

	First name	Family name		Signature	Date
1st author	Ruizhi	Yu	I agree to the proposed new authorship shown in section 4	Rui Zhi Yu	2025.12.19
2nd author	Yunfei	Yan	I agree to the proposed new authorship shown in section 4	Yun fei Yan	2025.12.19
3rd author	Junjie	Lin	I agree to the proposed new authorship shown in section 4	Junjie Lin	2025.12.19
4th author	Hucai	Li	I agree to the proposed new authorship shown in section 4	Hucai Li	2025.12.19
5th author	Youqing	Cai	I agree to the proposed new authorship shown in section 4	Youqing Cai	2025.12.19
6th author	Lixin	Wang	I agree to the proposed new authorship shown in section 4	LiXin Wang	2025.12.19
7th author	Xusheng	Liu	I agree to the proposed new authorship shown in section 4	Xusheng Liu	2025.12.19

SPRINGER NATURE Springer Nature is one of the world's leading global research, educational and professional publishers, created in May 2015 through the combination of Nature Publishing Group, Palgrave Macmillan, Macmillan Education and Springer Science+Business Media.

Change of authorship request form - Journals (pre-acceptance)

	First name	Family name		Signature	Date
8th author	Zhiren(CA)	He	I agree to the proposed new authorship shown in section 4	Zhiren He.	2025.12.19

Please use an additional sheet if there are more than 10 authors.

In case of author collaborations with formal agreement:

	Name of consortium/consortia	First name	Family name		Signature	Date
Representative/legal guarantor				I agree to the proposed new authorship shown in section 4 **/and the addition/removal*of my name to the authorship list** /and the proposed change in corresponding author		

Both added/removed authors should complete the information in the first table under Section 6.

---- End of form ----

SPRINGER NATURE Springer Nature is one of the world's leading global research, educational and professional publishers, created in May 2015 through the combination of Nature Publishing Group, Palgrave Macmillan, Macmillan Education and Springer Science+Business Media.
